# Predictive Genetic Biomarkers for the Development of Peritoneal Metastases in Colorectal Cancer

**DOI:** 10.3390/ijms241612830

**Published:** 2023-08-15

**Authors:** Danique J. I. Heuvelings, Anne G. W. E. Wintjens, Laura Moonen, Sanne M. E. Engelen, Ignace H. J. T. de Hingh, Liselot B. Valkenburg-van Iersel, Marcel den Dulk, Jan Beckervordersandforth, Sharon G. M. Thijssen, Daphne J. G. Leunissen, Laurents P. S. Stassen, Daniel Keszthelyi, Zlatan Mujagic, Ernst-Jan M. Speel, Nicole D. Bouvy

**Affiliations:** 1 NUTRIM School of Nutrition and Translational Research in Metabolism, Maastricht University, 6229 ER Maastricht, The Netherlands; 2 Department of General Surgery, Maastricht University Medical Center (MUMC+), 6229 HX Maastricht, The Netherlands; 3 GROW School for Oncology and Reproduction, Maastricht University, 6229 ER Maastricht, The Netherlands; 4 Department of Pathology, Maastricht University Medical Center (MUMC+), 6229 HX Maastricht, The Netherlands; 5 Department of General Surgery, Catharina Ziekenhuis, 5623 EJ Eindhoven, The Netherlands; 6 Department of Internal Medicine, Division of Medical Oncology, Maastricht University Medical Center (MUMC+), 6229 HX Maastricht, The Netherlands; 7 Department of Surgery and Transplantation, University Hospital RWTH Aachen, 52074 Aachen, Germany; 8 Division of Gastroenterology and Hepatology, Maastricht University Medical Center (MUMC+), 6229 HX Maastricht, The Netherlands

**Keywords:** colorectal cancer, peritoneal metastases, biomarkers, genetic mutations, next generation sequencing

## Abstract

Metastatic colorectal cancer (CRC) is a common cause of cancer-related mortality, of which peritoneal metastases (PMs) have the worse outcome. Metastasis-specific markers may help predict the spread of tumor cells and select patients for preventive strategies. This exploratory pilot study aimed to gain more insight into genetic alterations in primary CRC tumors, which might be a predictive factor for the development of PM. Forty patients with T3 stage CRC were retrospectively divided in three groups: without metachronous metastases during 5-year follow-up (M0, *n* = 20), with metachronous liver metastases (LM, *n* = 10) and with metachronous PM (PM, *n* = 10). Patients with synchronous metastases were excluded. Primary formalin-fixed paraffin-embedded tumor samples were analyzed via comprehensive genome sequencing (TSO500 analysis) to identify DNA alterations and RNA fusion transcripts in 523 genes and 55 genes, respectively. Thirty-eight samples were included for final analysis. Four M0 tumors and one PM tumor were microsatellite instable. *BRAF* mutations were uniquely identified in three microsatellite-stable (MSS) PM tumors (37.5%, *p* = 0.010). RNA analysis showed an additional *FAM198A-RAF1* fusion in one PM sample. *BRAF* p.V600E mutations were only present in PM patients with MSS tumors. Greater attention should be paid to BRAF-mutated tumors in relation to the development of metachronous PM.

## 1. Introduction

Metastatic colorectal cancer (CRC) is a common cause of cancer-related mortality. At initial diagnoses, almost one-fourth of CRC patients present with metastases [1,2]. Liver metastases (LMs) occur most frequently, followed by peritoneal metastases (PMs) [2,3]. PMs are characterized by the development of solid tumor deposits on the peritoneal surface [4]. It is suggested that PMs develop through the shedding of tumor cells from the primary tumor, leading to intraperitoneal seeding [1]. Synchronous PMs are found in approximately 5–15% of patients with colorectal cancer at primary surgery [2,3,4,5], but PM may also develop metachronously after curative-intent treatment of the primary tumor. In clinical studies, these metachronous PMs are reported in 4–12% of patients following curative resection for colon cancer and in 2–19% of patients following curative resection for rectal cancer [5]. Routine imaging techniques frequently fail to detect PMs due to their small size along with the inherently low contrast resolution of the soft tissue in which they occur, resulting in an underestimation of their true incidence [2,5,6].

Since colorectal PMs occur less frequently than liver and lymph node metastases, they are considered less important from a prognostic perspective [7,8]. Nonetheless, the consequences of PMs are significant. Without treatment, the average life expectancy is six to twelve months after diagnosis [4,9,10]. Currently, the only potential treatment to improve the survival of patients with colorectal PM is the surgical removal of all visible tumor deposits (cytoreductive surgery, CRS) followed by the application of heated chemotherapy, called hyperthermic intraperitoneal chemotherapy (HIPEC). Inquiries emerged concerning the requisite of adjuvant HIPEC subsequent to CRS, as CRS alone resulted in a survival advantage of over 40 months in the PRODIGE-7 trial [11]. 

Unfortunately, only a selection of physically fit patients with limited colorectal PM (peritoneal cancer index (PCI) below 20) are eligible for this therapy [2,9,10,12]. With the changing perspective of this disease, many aspects of the biological and clinical understanding of this challenging disease process remain to be better understood [13]. 

In patients with synchronous PMs, genetic alterations are interesting as a biomarker to determine prognosis or to predict response to therapy [14,15,16]. In addition, genetic alterations in the primary tumor may also be useful for the prediction of PM occurrence. Several pathogenic mutations occur during adenoma-to-carcinoma transformation in CRC. Important oncogenes are adenomatous Polyposis Coli (*APC*), tumor suppressor gene *TP53*, *KRAS*, transforming growth factor beta (*TGF-β*), phosphatidylinositol-4,5-bisphosphate 3-kinase catalytic subunit alpha (*PIK3CA*), and loss of the chromosome arm 18q [17]. Additionally, some genetic alterations are described in relation to a specific metastatic site. For example, differences in *APC*, *BRAF*, *KRAS,* and *NRAS* are associated with the location of the primary tumor, whereby mutations in *KRAS* and *BRAF* seem to result in worse overall survival and the recurrence site in patients with PM [17,18]. 

The aim of this study was to identify genomic changes in primary CRC that are associated with development of PMs, which would allow early detection and personal and early patient treatment. Such a study has not been reported yet [17,19], despite the growing attention and possibilities for the genomic analysis of cancer using, for example, next-generation sequencing (NGS) techniques with broad gene panels investigating DNA and RNA alternations.

In this explorative study, we identified specific DNA/RNA alterations (via TruSight Oncology (TSO) 500 analysis) in primary colorectal T3 tumors to predict metachronous PMs after curative resection.

## 2. Results

### 2.1. Study Cohort

Initially, 40 cases were selected according to predefined in- and exclusion criteria. After revision of the CT, one of the patients was diagnosed with a synchronous metastatic lesion in the lung and excluded from further analysis. All patient characteristics and clinicopathological variables are summarized in Table 1. Most patients were males (64%), with a median age of 69 years (61.00–74.00) at the time of diagnosis of the primary colorectal tumor. There was an overall significant difference for differentiation grade (*p* value < 0.001) and neoadjuvant treatment (*p* value = 0.039). After pairwise comparison, a significant difference was found in the differentiation grade when primary tumors of patients with metachronous PM were compared to patients without metachronous metastases (M0) and with metachronous liver metastases (LM) (*p* value < 0.001 and 0.015, respectively). Patients in the LM group were more often treated with neoadjuvant therapy compared to the M0 group (*p* value = 0.030), which did not remain significant after Bonferroni correction.

### 2.2. DNA Sequencing

In one LM sample, no (likely) pathogenic mutations were found, most probably due to the low residual tumor area after neo-adjuvant treatment. This outcome was considered unreliable, and the sample was excluded from further DNA analysis. The final study cohort thus consisted of 38 patients (Supplementary Section S1, Figure S1 and Table S1).

Microsatellite instability (MSI) analysis showed that a total of 5/38 samples (four M0 [20%] and one PM [11%]) were MSI with a median of 53.91% unstable MSI sites (Q1 32.55–68.11; Supplementary Section S1, Figure S2). These samples also showed a significant TMB with a median of 64.3 mut/Mb (Q1 49.45–Q3 180.60). All significant MSI and TMB patients had a right-sided primary tumor with poor or poor/moderate differentiation grade. The occurrence of MSI and TMB was not significantly different between the three groups. One of the MSI samples harbored a nonsense mutation in *MSH6* (i.e., c.3772C>T p.(Q1258*)), a DNA mismatch repair protein, which could explain the instability of the sample. All other four samples showed *MLH1* promotor hypermethylation. 

Mutational signatures from each sample were individually analyzed. Base substitution of C>T and T>C were the most common ones in all samples. No specific profile was identified when comparing the three subgroups. A general overview of all variant type frequencies and amplifications is displayed in Supplementary Section S1, Table S2 and of all tumor mutations and amplifications in Table S3. Analysis of the total cohort did not identify significant gene mutations in the PM group nor other subgroups. As MSI samples showed a lot of passenger genes that were influencing analysis outcomes, all MSI samples were excluded for a separate analysis with only microsatellite stable (MSS) tumors. The analysis of the total cohort (MSI + MSS samples, *n* = 38) can be found in Supplementary Section S2 (Figures S4 and S5, Table S5).

#### 2.2.1. MSS Samples Analysis

**Figure 1:**
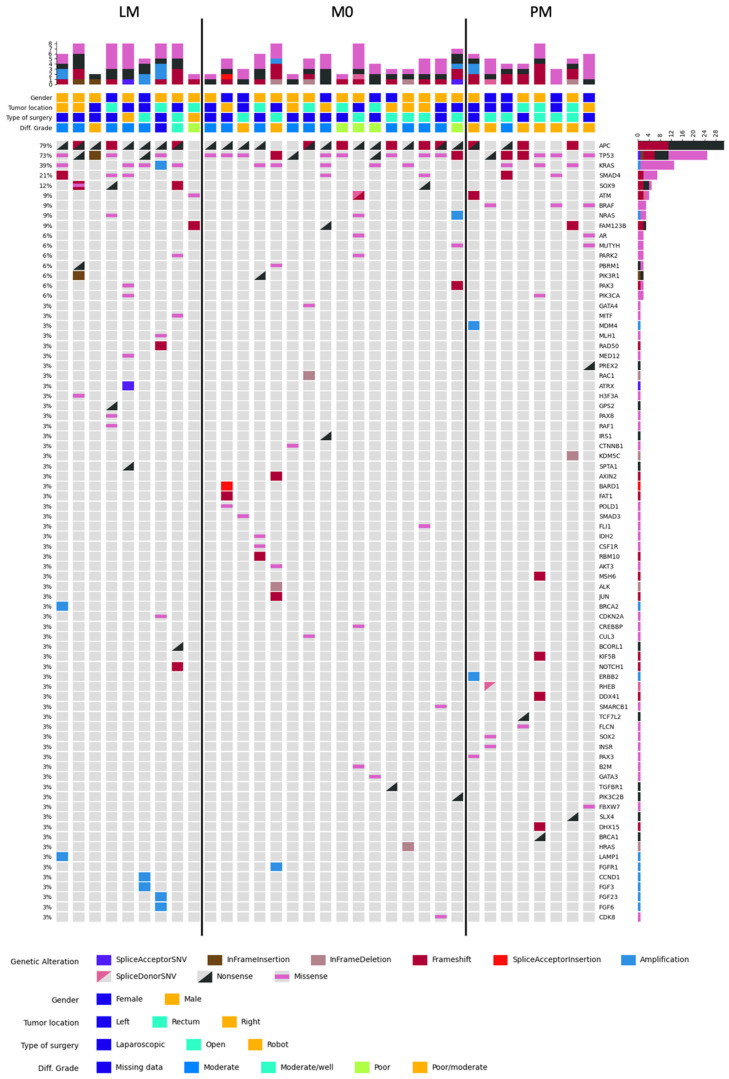
Oncoplot of variants across MSS samples. Genes on *y*-axis; samples on *x*-axis.

**Figure 2:**
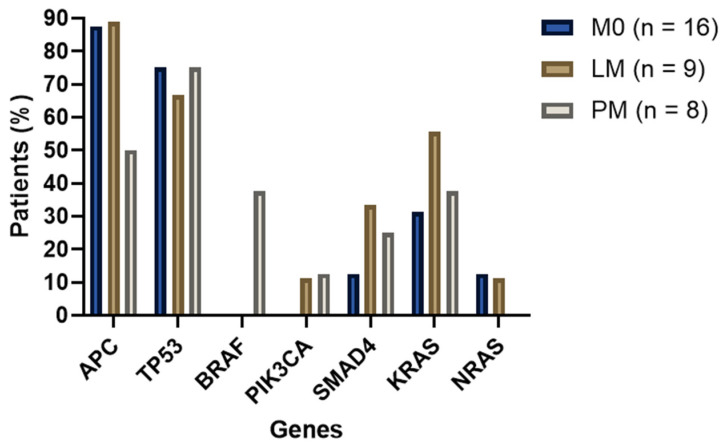
Distribution of well-known cancer genes related to MSS CRC.

All MSI tumors were excluded for a separate analysis. This resulted in a study population of 33 patients with MSS tumors (M0 N = 16, LM N = 9, and PM N = 8). A total of 164 (likely) pathogenic genetic alterations were detected in 78 genes (Figure 1). Missense, frameshift, and nonsense mutations were most commonly detected. When comparing the occurrence of all variant types, no significant differences were found. The distribution among cancer genes related to CRC was investigated (Figure 2). APC mutations occurred most frequently; in 4/8 (50%) of the PM cases and 8/9 (89, 89%) LM and 14/16 (87, 50%) M0 patients (not significant). *BRAF* (c.1799T>A p.(V600E) exon 15) mutations were only present in PM patients in this cohort (3/8 = 37.5%, *p* value = 0.010). None of the M0 samples were carrying *PIK3CA* mutations after MSI exclusion, and none of the PM samples were carrying *NRAS* mutations, although these findings were not significantly different. A detailed overview of all MSS subgroup comparisons with statistical *p* values can be found in the Supplementary Section S1, Table S4.

#### 2.2.2. Additional Analyses 

Pathways, molecular functions, and biological processes were not significantly different between the three CRC subgroups. Also, after the additional inclusion of all identified variants of uncertain significance (VUSs), no significant differences were found between the subgroups. A detailed overview of all additional data analyses can be requested via the corresponding author.

### 2.3. RNA Sequencing

**Figure 3:**
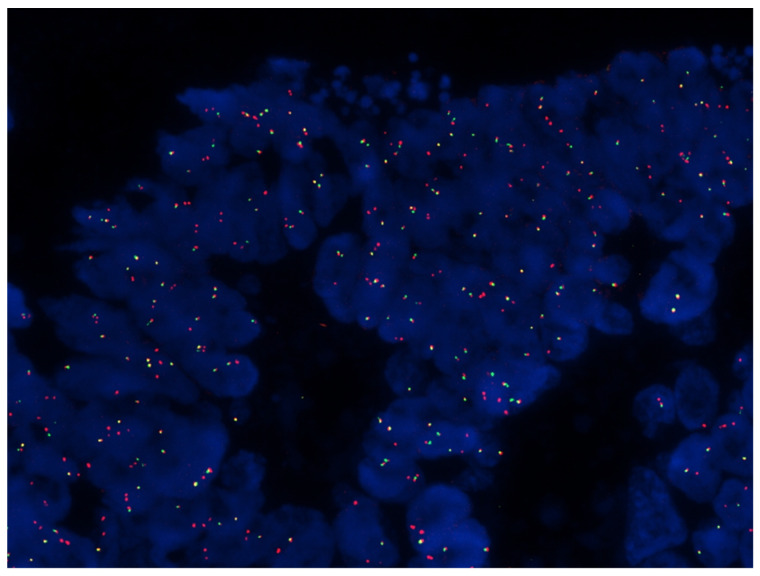
FISH analysis of the M0 sample harboring the *TARSL2—NTRK3* fusion, showing isolated green and red signals confirming an *NTRK3* gene rearrangement.

RNA sequencing was performed on 28 samples, divided as follows: M0 (*n* = 10), LM (*n* = 9), and PM (*n* = 9). Data analysis revealed no splice variants for the genes in the panel, whereas three samples (one M0 and two PM samples) showed gene fusion transcripts, which are summarized in Table 2. Interestingly, two gene fusions were identified which can be considered driving mutations, i.e., *FAM198A*-*RAF1* and *TARSL2*-*NTRK3*. The *NTRK3* fusion was confirmed via fluorescence in situ hybridization (FISH), using an *NTRK3* break-apart probe (Figure 3).

## 3. Discussion

In this study, we performed an integrated pan-cancer oncology enrichment next-generation sequencing assay (TSO500 analysis) to assess DNA and RNA alterations in 523 and 55 genes, respectively, in primary colorectal adenocarcinomas with or without metachronous PM or LM. Our cohort showed a significant difference in differentiation grade when PM samples were compared to LM and M0 samples, and in the LM group for neoadjuvant treatment. Genetic analysis of all MSS tumors revealed that pathogenic *BRAF* exon 15 p.(V600E) mutations were exclusively identified in three *RAS* wildtype tumors with metachronous PM (37.5%, *p* value = 0.010). RNA sequencing identified a *FAM198A*-*RAF1* fusion in an additional tumor with PM, as well as a *TARSL2*-*NTRK3* fusion in a M0 sample. 

### 3.1. Patient Characteristics and Clinicopathological Variables

We identified two clinicopathological characteristics that were significantly different between the three tumor groups. First, the PM group contained more poor/moderately differentiated tumors, while M0 and LM tumors were more often moderately differentiated. The latter was also shown in an extensive analysis of the association between metachronous PM and clinicopathological characteristics by Zhang et al. [20]. Tumor location is not mentioned in this analysis, although another study reports that right-sided primary colorectal tumors are associated with PM [2]. Only 22% of the PM tumors in our cohort were right sided. Second, the lowest tumor cell percentages were observed in the LM group, which may be explained by the fact that in this group, more patients received neoadjuvant treatment via chemoradiation because of a low rectal primary origin. Other previously described clinicopathological risk factors for the development of metachronous PMs are advanced tumor stage, infiltrative or ulcero-infiltrative tumors, a history of perforation, and obstruction [1,4,21]. A clinical trial investigating the potential of adjuvant HIPEC in high-risk PM patients, based on these clinicopathological risk factors, showed that adjuvant HIPEC did not improve survival as compared to patients receiving systemic adjuvant chemotherapy [22]. In contrast, Arjona-Sánchez et al. concluded that adjuvant HIPEC therapy might be useful in patients with T4 tumors [23]. These outcomes suggest that specific biomarkers identified in the primary tumor might be helpful to further estimate the risk of metastatic spread and the need for preventive adjuvant treatments. As our study population has a semi-advanced tumor stage (T3) without (ulcero-)infiltrative or obstructing tumors, we exclude any influence of these possible clinical–pathological risk factors in our current study. 

### 3.2. DNA and RNA Sequencing

The most frequently mutated cancer genes found in our study include *APC*, *TP53*, *KRAS, SMAD, NRAS, BRAF, PIK3CA*, and *SOX9*. These genes are well known to be involved in the tumorigenesis of CRC [17]. Prevalence data in the literature on these well-known oncogenes are in line with our findings [15,24,25,26,27,28,29,30,31,32,33,34,35,36,37,38,39,40,41,42,43,44,45]. In addition, 12.5% of our tumors contained MSI, in four cases associated with MLH1 promoter hypermethylation, and in one case with an inactivating MSH6 mutation. This finding is in accordance with the literature, as was the finding that these tumors are often right-sided [46,47,48]. MSI results from the inactivation of the mismatch repair genes (MMR), which leads to the accumulation of somatic mutations, genomic instability, and cancer-associated alterations [31]. TMB represents the total number of mutations per Mb found in the DNA of tumor cells and is therefore often significantly higher in MSI tumors. In this study, the five tumors with MSI all had a high TMB (IQR 49.45–Q3 180.60). 

It has been suggested that MSI status may be useful as a predictor of the risk of developing metachronous CRC, because it can cause a further increase in metastatic potential [24,46]. However, we did not observe a higher incidence of MSI tumors in our CRC cohort that developed metastases. Interestingly, *BRAF* p.V600E mutations were found to be exclusively present in PM patients with *RAS* wildtype MSS tumors (37.5%, *p* value = 0.010). Approximately 10–14% of all CRC cases have *BRAF*-activating mutations [24,31,49]. *BRAF* encodes a serine/threonine protein kinase, which plays an important role in the mitogen-activated protein kinase (MAPK) pathway. This pathway drives cell proliferation, differentiation, migration, survival, and angiogenesis, and therefore, changes in this pathway are associated with tumorigenesis [49]. The *BRAF* p.V600 mutation, caused by a transversion in exon 15 resulting in a valine amino acid substitution [50], accounts for more than 90–95% of *BRAF* mutations [31,49] and is associated with poor overall survival [24]. In addition, we identified a *FAM198A*-*RAF1* fusion in one PM sample. Both RAF1 and BRAF belong to the *RAF* family of protein kinases playing a role in MAPK signaling. Previous studies suggested that *BRAF* p.V600 mutant tumors are more likely to develop PM [15,32,35,36,37,51]. Therefore, we and some authors recommend analyzing *BRAF* mutation for its prognostic value in primary T3 CRC [31,34].

The clinical significance of *NTRK3* fusion identified in our study, in the setting of CRC, as well as the possibility for targeted treatments should be explored in the future.

Prior to our explorative study, we performed a systematic review to summarize the current knowledge on genetics and genomics in CRC-PM [19]. An NGS analysis with 409 cancer genes showed several additional genetic mutations, i.e., *ARID1A*, *PKHD1*, *UBR5*, *PAX5*, *TP53*, *ASXL1*, and *AR*, presumably associated with PM [52]. In our TSO500 NGS panel, *ARID1A*, *PAX5*, *TP53*, *ASXL1*, and *AR* were included as well. *AR* and *ARID1A* mutations occurred in one PM (11%) and one M0 sample (5%). Only one *PAX5* mutation was found in one M0 patient, and *ASXL1* mutations were not detected. Thereby, the suggested genes related to PM by Lee et al. are not confirmed in our paper. The latter may be explained by the difference in study population; Lee et al. included patients with small obstructing adenocarcinomas (≤3 cm) with synchronous or metachronous PM and compared them with large non-obstructing tumors without PM. Another explanation could be our small sample size. Other authors describe *NEK2*, *MACC1*, *REG1A*, *KIF18A*, *RET*, and *TIP60* as possible PM-related cancer genes [53,54,55,56,57,58]. In our TSO500 panel, only *RET* was investigated. In contrast to the suggestion of Yang et al. concerning the association of *RET* mutations and PM, we did not identify any mutation in this gene in our cohort [57].

Another factor that can contribute to the difficulty of finding biomarkers is the genetic differences between the primary tumor and metastatic lesions. Studies investigating the differences between peritoneal lesions and their primary tumors reported some small unique differences [59], whilst other studies report high concordance [60,61,62]. A very recent study by Lenos et al. showed that peritoneal lesions seemed to have much more similarity to their primary tumor compared to other metastases, and these lesions seemed to retain both clonal heterogeneity and transcriptional profile [61].

A new way to look at CRC tumors is through dividing them into subtypes, for example, the previously described four consensus molecular subtypes (CMS 1–4). These subtypes aid in prognostication as well as in determining treatment strategies for individual patients based on the mutations, activated pathways, and phenotypic characteristics and responses to treatment of other tumors with similar signatures [63]. The majority of PMs in their study are of the CMS4 subtype, known as the mesenchymal subgroup [64,65]. CMS4 is presented in 23% of CRC cases, which are most often distal tumors with poor relapse-free and overall survival and harbor prominent transforming growth factor β activation, stromal infiltration, and angiogenesis [66,67]. CMS4 tumors have extremely low levels of hypermutation, MSS, and very high somatic copy number alteration counts [63]. The latter was also seen in our cohort. Unfortunately, we were not able to examine all of these characteristics in our study due to the limited content of our RNA NGS gene panel. Therefore, the translation to CMS subgroups was not possible in our study. 

### 3.3. Treatment Options and Future Perspectives

*BRAF* mutations can be considered as an independent negative prognostic factor in early stage MSS tumors and as a negative predictive factor for therapeutic approaches [49]. The therapeutic approach to treat *BRAF*-mutated tumors is not straightforward due to its resistance to standard therapies [49]. Research into anti-epidermal growth factor receptor (EGFR) and anti-vascular endothelial growth factor (VEGF) antibodies has not shown statistical benefits in *BRAF*-mutated patients [49,68]. *BRAF* inhibitors (i*BRAF*) have revolutionized the treatment of *BRAF* V600E metastatic melanoma, but so far, results in CRC patients are disappointing due to resistance [34,49,69]. Studies are currently ongoing with dual or triple drug therapy to blockade the *MAPK* pathway [49,69]. Until now, partial activity of different combinations has been shown, but this is far from the promising results in melanoma patients. Ongoing research will hopefully demonstrate that combination strategies with *iBRAF* and other drugs can overcome the lack of efficacy [49]. As survival is about half as long as that of *BRAF* wildtype patients [68], there is an urgency to unravel new treatments that improve *BRAF*-mutant CRC patients’ outcomes.

In current clinical practice, the classification of the MSI status is the only genetic test that is routinely performed in CRC patients to decide adjuvant therapy decisions [70]. Other genetic tests, such as *BRAF* mutation status, are only evaluated in metastatic tumors. Based on the results of this paper, we believe greater attention should be paid to *BRAF*-mutated tumors in relation to the development of metachronous PM in CRC patients without metastases. Standard clinical screening for *BRAF* mutations might feel too early as it does not offer any new treatment options, but a stricter follow-up in this population may be clinically beneficial. Based on new international guidelines, the first follow-up CT scan is not performed until 12 months after primary surgery. However, in a *BRAF*-mutated population, earlier follow-up imaging and more clinical monitoring for PM development may be warranted. Of course, future prospective research (e.g., with liquid biopsies) into the validation of *BRAF* mutations in relation to the development of metachronous PM is needed to substantiate this proposition.

### 3.4. Strengths and Limitations

A very homogenous group of tumors was selected for genetic analysis. To our knowledge, this is the first study investigating T3 tumors in relation to metachronous CRC metastases. Previous studies focused on T4 tumors with mostly synchronous PMs and had no other metastases group (LM) as a comparator. While PMs may develop from different cancer types, we specifically examined the colorectal origin and excluded appendiceal origin as it is known that gene expression from appendiceal tumors is distinct from CRC [59]. Due to refinements in DNA and RNA extraction techniques from formalin-fixed paraffin-embedded (FFPE) tissue material, the sensitivity of DNA and RNA testing has been increased. Our targeted TSO500 NGS technique accurately measures TMB, microsatellite instability, single-nucleotide variants, indels, copy-number/structural variation, and gene fusions in a single assay using relatively small amounts of DNA and RNA as input. Combining DNA and RNA hybrid-capture with sophisticated informatics reduces errors and yields high-quality data, even from FFPE samples. 

We did not perform an extensive sample size calculation due to the predictive and explorative character of this study. Despite the efforts made to create as much homogeneity between the three groups as possible, the number of patients in our cohort is small. A larger-scale study should be conducted to confirm the mutation differences in relation to PMs. Thereby, being a retrospective study, there is a likelihood of selection bias and information bias. Additionally, we performed a very broad cancer gene analysis with our TSO500 panel, although the method does not cover all genes. Through performing whole exome or genome sequencing (WES or WGS), potential candidate genes that can act as a predictive PM biomarker that are not included in the TSO500 panel may be identified. Unfortunately, WES and/or WGS are more expensive and have additional logistic limitations. 

## 4. Materials and Methods

This study was conducted in a collaboration between the Maastricht University Medical Centre (MUMC+) and Catharina Hospital Eindhoven (CZE). The study was approved by the Institutional Medical Ethics Committee from MUMC+ (nr. 2021-2888) and CZE (nr. 2021-089) and conducted according to the Declaration of Helsinki. 

### 4.1. Patients 

The medical records of patients who underwent curative resection between 1 January 2012 and 31 December 2021 for colorectal adenocarcinoma were retrospectively reviewed. The research team deliberately chose to include a maximum of 40 patients in this pilot study, based on clinical prediction modeling which states at least 10 persons with the event (development of PM or LM) and 10 persons without the event (no metastases within 5 years) per included variable in the prediction model to obtain sufficient power [71]. Patients with T3 tumors were classified into three groups: those who had developed metachronous PMs (*n* = 10); those who had developed metachronous liver metastases (LM, *n* = 10); and those who never developed metastatic disease within 5 years after primary surgery (M0, *n* = 20). Patients with metachronous PMs were not allowed to be diagnosed with metachronous LMs and vice versa. As T4 tumors penetrate the surface of the visceral peritoneum and directly invade other organs or structures, the risk of spread into the peritoneal cavity is higher. Therefore, we only included T3 tumors and deliberately excluded T1 and T2 tumors to create a homogenous population. Patients with synchronous disease were excluded. Patients in the LM and PM group had no signs of metastases during resection of the primary tumor but were diagnosed with PM or LM during follow-up, at least 6 months after initial surgery. Patients in the M0 group did not develop any type of metastases during the follow-up period of at least 5 years. All in- and exclusion criteria are summarized in Table 3. Patient record files were screened, and the first 40 patients who met inclusion criteria were contacted. Informed consent was obtained from all participants. Demographics, pre-operative, operative, and follow-up data of all patients were retrospectively retrieved from medical records.

### 4.2. Tumor Samples

Primary tumor FFPE tissue samples were obtained from MUMC+ and CZE. From each FFPE tissue specimen, 10 paraffin sections of 5 μm were cut. Hematoxylin and eosin (H&E) staining was performed. An experienced pathologist (J.B.) marked the tumor circumflex and estimated the tumor cell percentage under the microscope. Only samples with ≥10% tumor cell percentage were considered eligible for further analysis. Subsequently, microdissection with a pointed surgical blade was performed.

DNA and RNA were extracted and isolated using a Maxwell RSC^®^ System for Genomic DNA or RNA Extraction with a FFPE AS1450 kit and FFPE AS1440 kit, respectively (Promega, Madison, WI, USA). A blank control sample was analyzed in parallel to each set of samples. A minimum amount of 40 ng DNA or RNA was necessary for further analysis. DNA samples were stored at 4 °C and RNA samples at −80 °C. Fragment analysis of both DNA and RNA samples was performed as quality control. For DNA, a PCR was performed to visualize all DNA fragments. For RNA, the samples were assessed using a 4150 TapeStation system, which separates nucleic acids through electrophoresis. All fragments needed to be at least 200 bp in length.

### 4.3. TruSight Oncology 500 Analysis

TSO500 is an NGS assay that enables the comprehensive genomic profiling of tumor samples. The TSO500 panel (20028216; Illumina, Hayward, San Diego, CA, USA) was used to detect mutations and identify other relative pan-cancer genes in the tumor samples, as previously described by Verkouteren et al. [72]. The analysis includes 523 genes for mutations (all for single-nucleotide variants (SNVs)) and 59 for copy number variations (CNVs) (amplifications, insertions, and deletions). In addition, the assay allows for the identification of MSI and TMB. Besides DNA analysis, 55 genes are screened for fusion and splice variants on the RNA level. All genes included in the TSO 500 panel can be found in Supplementary Section S1, Figure S3. DNA and RNA processing and the generation of library preparations were performed according to the manufacturers’ instructions. Data analysis was performed using the TSO500 Local App (Illumina, Hayward, San Diego, CA, USA). For DNA analysis, additional thresholds were maintained. First, for variant allele frequency, a percentage of ≥5% was maintained. Second, for classification as an amplification, a fold change of ≥3 was maintained. Third, the threshold for classification as MSI-high was ≥20% of microsatellite sites being unstable. Fourth, a threshold of ≥15 mutations per megabase (mut/Mb) was used to define high TMB. Variants were classified subsequently using the inline Varsome application (access via https://varsome.com). Only pathogenic and likely pathogenic variants were included for further analysis; variants of uncertain significance (VUSs) were excluded. 

### 4.4. Statistical Analysis

Gene mutation frequencies and associations between the found mutations and pathological patient characteristics were estimated. Analysis of both the total cohort (with MSI samples) as well as MSS samples only were performed. Numerical variables were presented as medians with interquartile range (IQR) as appropriate. For categorical variables, the number of patients and percentage were used. To evaluate the statistical significance of numerical variable differences observed between groups, non-parametric tests (Kruskal–Wallis and Mann–Whitney U-tests) were applied. Differences in categorical variables were tested using the Fisher–Freeman–Halton test and Fisher’s exact test. Bonferroni correction for multiple comparison was applied to significant outcomes. All tests were two-sided, and differences were considered significant when the *p* value was <0.05. All the statistical analyses were performed with SPSS (IBM SPSS Statistics for Apple, Version 27, Armonk, NY, USA). In addition, an analysis with protein analysis through evolutionary relationships (PANTHER) was performed [73]. The latter was performed for Gene Ontology molecular functions and biological processes [74,75], for PANTHER pathways, and for Reactome pathways [76]. For each sample, the significantly enriched terms were extracted for subsequent analyses using R (R core team, version 4.2.0., Vienna, Austria). Analysis and visualization of the genetic outcomes were carried out with Python (Matplotlib v3.7.0, Salt Lake, UT, USA) and GraphPad Prism (GraphPad software for Apple, version 8.0.0, San Diego, CA, USA).

## 5. Conclusions

Over the last decade, the genetic analysis of CRC has evolved enormously, resulting in better tumor classifications, improved treatment decisions, and finally enabling personalized treatment options. Specific genetic changes and mutations that could predict PM remain largely unknown. In our cohort, we identified genes that have not been described in relation to metachronous PMs, or metastases in general, before. The clinical significance of this finding remains unknown due to the small sample size. *BRAF* V600E mutations were only present in PM patients with MSS tumors. We believe greater attention should be paid to *BRAF*-mutated tumors in relation to the development of metachronous PMs. Future prospective research into and validation of the molecular players identified here, specifically within non-synchronous tumors, might influence the efficacy of existing and future diagnostic (biomarker identification), prognostic (patient grouping and recurrence), and therapeutic (molecular) actions.

## Figures and Tables

**Table 1:** Comparison of patient characteristics and clinicopathological variables in the relation to the development of metastases.

Variable	M0 (N = 20)	LM (N = 10)	PM (N = 9)	*p* Value
**Age at time of diagnosis** (years)—median (Q1–Q3)	69.00	69.00	68.00	0.801 ^a^
	(62.00–74.90)	(63.75–74.25)	(58.00–74.00)	
**Gender**–*n* (%)				0.514 ^b^
Male	12 (60)	8 (80)	5 (55.6)	
Female	8 (40)	2 (20)	4 (44.4)	
**Primary tumor location** ^†^—*n* (%)				0.433 ^b^
Right colon	10 (50)	2 (20)	2 (22.2)	
Left colon	7 (35)	5 (50)	4 (44.4)	
Rectum	3 (15)	3 (30)	3 (33.3)	
**Tumor size** (cm) —median (Q1–Q3)	4.10	2.25	3.00	0.061 ^a^
	(3.28–5.38)	(1.80–5.43)	(2.40–3.50)	
**Differentiation grade**—*n* (%) *				**<0.001** ^**b**^
Poor	4 (20)	0 (0)	0 (0)	
Poor/moderate	2 (10)	2 (20)	8 (88.9)	
Moderate	14 (70)	6 (60)	1 (11.1)	
Moderate/well	0 (0)	1 (10)	0 (0)	
**Type of surgery**—*n* (%)				0.153 ^b^
Open	10 (50)	2 (20)	5 (55.6)	
Laparoscopic	10 (50)	6 (60)	4 (44.4)	
Robot assisted	0 (0)	2 (20)	0 (0)	
**Positive lymph nodes**—*n* (%)				0.389 ^b^
No	11 (55)	8 (80)	5 (55.6)	
Yes	9 (45)	2 (20)	4 (44.4)	
**Neoadjuvant treatment**—*n* (%)				**0.039** ^**b**^
No	17 (85)	4 (40)	7 (77.8)	
Yes	3 (15)	6 (60)	2 (22.2)	
**Adjuvant treatment**—*n* (%) *				0.247 ^b^
No	9 (45)	7 (70)	4 (44.4)	
Yes	11 (55)	2 (20)	5 (55.6)	
**Oncological history**—*n* (%)				0.882 ^b^
No	18 (90)	8 (80)	8 (88.9)	
Yes	2 (10)	2 (20)	1 (11.1)	
**Oncological family history**—*n* (%) *				1.000 ^b^
No	6 (30)	3 (30)	0 (0)	
Yes	12 (60)	5 (50)	1 (11.1)	
**Time between surgery and metastases** (months)—median (Q1–Q3)	N/A	18.09	16.42	0.744 ^c^
		(7.77–28.95)	(9.71–25.05)	
**PCI score**—median (Q1–Q3)	N/A	N/A	3.50	N/A
			(3.00–4.00)	

^a^ Kruskal–Wallis Test; ^b^ Fisher–Freeman–Halton Exact Test; ^c^ Mann–Whitney test. ^†^ Right-sided = from caecum to transverse colon; left-sided = from the splenic flexure to sigmoid. * Missing data in differentiation grade (LM = 1), adjuvant treatment (LM = 1), and oncological family history (M0 = 2, LM = 2 and PM = 8). M0, no metastases; LM, liver metastases; PM, peritoneal metastases; Q1–Q3, quartile 1–quartile 3; N/A, not applicable; PCI, peritoneal cancer index.

**Table 2:** Detailed output of RNA analysis.

M Group	Gene Pair	Breakpoint 1	Breakpoint 2	Fusion Supporting Reads
M0	*TARSL2*-*NTRK3*	Exon 18 chr15:102197123	Exon 14 chr15:88576274	19
PM	*FAM198A*-*RAF1*	Exon not found chr3:43101459	Exon 3 chr3:12653448	85
PM	*RPS6KB1*-*HSF5*	Exon 1 chr17:57970685	Exon 3 chr17:56544340	21

M0, no metastases; PM, peritoneal metastases.

**Table 3:** In- and exclusion criteria for patient selection.

Inclusion Criteria	Exclusion Criteria
Tumor histological type defined as an adenocarcinoma Pathology report confirmed a radical resection with >15 lymph nodes Pathological T3 classified according to the TNM classification M0 group: Follow-up of 5 years without development of metastases	Acute colorectal surgery with blow-out or proven perforation Anastomotic leak after surgery Patients with hereditary CRC LM and PM group: Metachronous metastases > 6 months after primary surgery

M0: no metastases; LM, liver metastases; PM, peritoneal metastases.

## Data Availability

Data supporting this study are included within Supporting Materials. More information can be gained through contacting the corresponding author.

## Supplementary Materials

The supporting information can be downloaded at: https://www.mdpi.com/article/10.3390/ijms241612830/s1.
