# Supplementary material for: Predictive Genetic Biomarkers for the Development of Peritoneal Metastases in Colorectal Cancer
Source: Int J Mol Sci. 2023 Aug 15;24(16):12830. doi: 10.3390/ijms241612830 (PMC10454220; doi:10.3390/ijms241612830)
Supplement: Supplementary file 1 [file ijms-24-12830-s001.zip › ijms-2549806-supplementary.pdf]

Supplementary Predictive genetic biomarkers for the development of peritoneal metastases in colorectal cancer

Supplementary Section S1

Figure S1. Details patient selection and data analysis

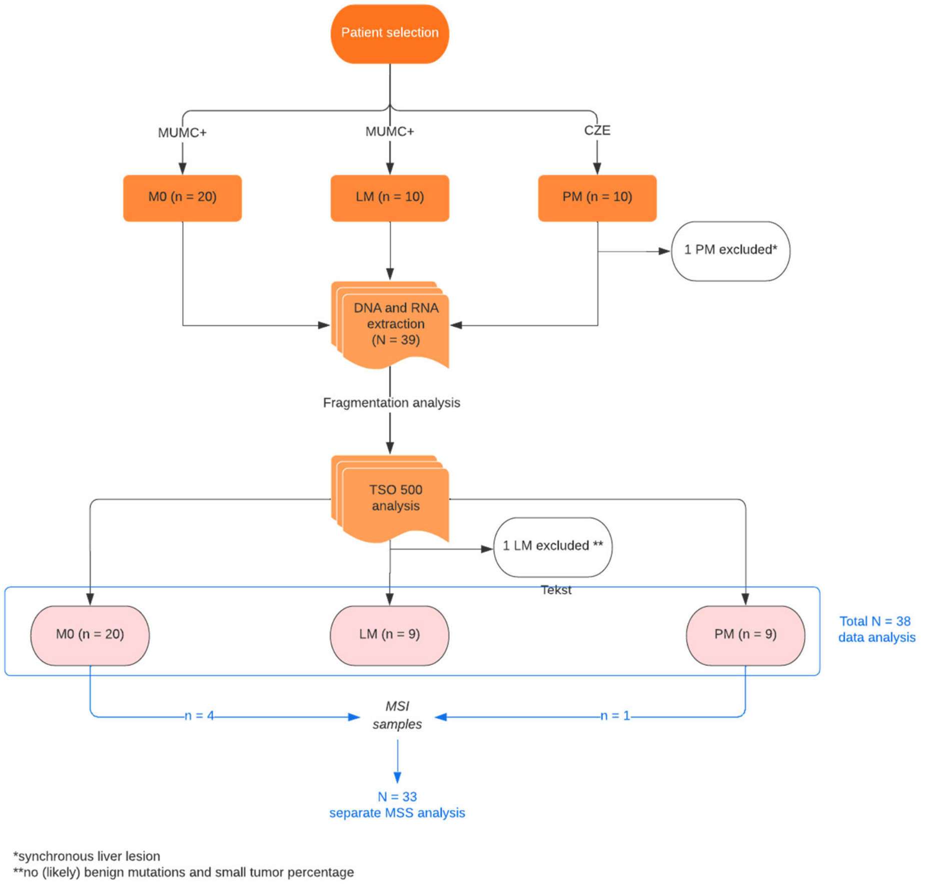

Figure S2. Boxplots of **(A)** median unstable MSI sites (%) and **(B)** median TMB.

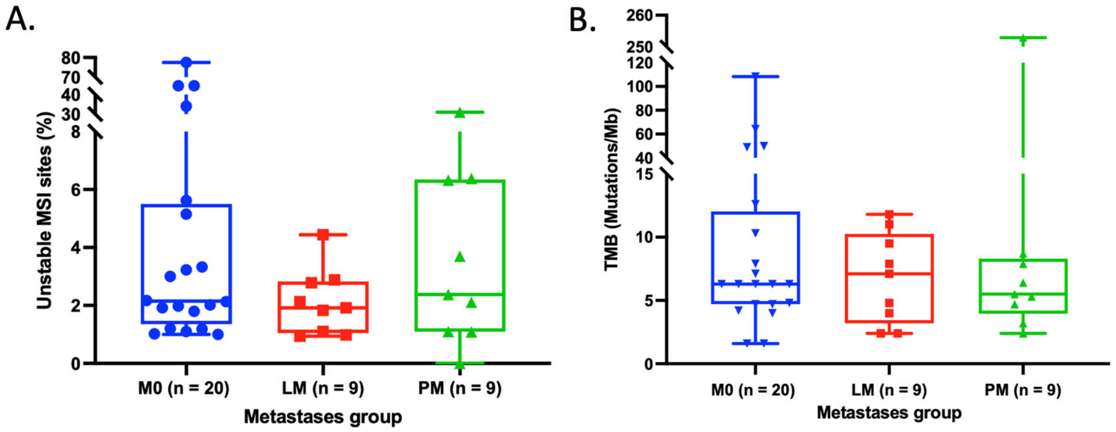

Figure S3. Overview of genes included in the TruSight Oncology 500 panel. [Illumina, Inc. (n.d.)].

					DNA content					
ABL1	*BRD4*	*CUX1*	*FAM175A*	*GATA6*	*IGF1*	*MAP3K13*	*NOTCH4*	*POLE*	*RPTOR*	*TAF1*
ABL2	*BRIP1*	*CXCR4*	*FAM46C*	*GEN1*	*IGF1R*	*MAP3K14*	*NPM1*	*PPARG*	*RUNX1*	*TBX3*
ACVR1	*BTG1*	*CYLD*	*FANCA*	*GID4*	*IGF2*	*MAP3K4*	*NRAS*	*PPM1D*	*RUNX1T1*	*TCEB1*
ACVR1B	*BTK*	*DAXX*	*FANCC*	*GLI1*	*IKBKE*	*MAPK1*	*NRG1*	*PPP2R1A*	*RYBP*	*TCF3*
AKT1	*C11orf30*	*DCUN1D1*	*FANCD2*	*GNA11*	*IKZF1*	*MAPK3*	*NSD1*	*PPP2R2A*	*SDHA*	*TCF7L2*
AKT2	*CALR*	*DDR2*	*FANCE*	*GNA13*	*IL10*	*MAX*	*NTRK1*	*PPP6C*	*SDHAF2*	*TERC*
AKT3	*CARD11*	*DDX41*	*FANCF*	*GNAQ*	*IL7R*	*MCL1*	*NTRK2*	*PRDM1*	*SDHB*	*TERT*
ALK	*CASP8*	*DHX15*	*FANCG*	*GNAS*	*INHA*	*MDC1*	*NTRK3*	*PREX2*	*SDHC*	*TET1*
ALOX12B	*CBFB*	*DICER1*	*FANCI*	*GPR124*	*INHBA*	*MDM2*	*NUP93*	*PRKAR1A*	*SDHD*	*TET2*
ANKRD11	*CBL*	*DIS3*	*FANCL*	*GPS2*	*INPP4A*	*MDM4*	*NUTM1*	*PRKCI*	*SETBP1*	*TFE3*
ANKRD26	*CCND1*	*DNAJB1*	*FAS*	*GREM1*	*INPP4B*	*MED12*	*PAK1*	*PRKDC*	*SETD2*	*TFRC*
APC	*CCND2*	*DNMT1*	*FAT1*	*GRIN2A*	*INSR*	*MEF2B*	*PAK3*	*PRSS8*	*SF3B1*	*TGFBR1*
AR	*CCND3*	*DNMT3A*	*FBXW7*	*GRM3*	*IRF2*	*MEN1*	*PAK7*	*PTCH1*	*SH2B3*	*TGFBR2*
ARAF	*CCNE1*	*DNMT3B*	*FGF1*	*GSK3B*	*IRF4*	*MET*	*PALB2*	*PTEN*	*SH2D1A*	*TMEM127*
ARFRP1	*CD274*	*DOT1L*	*FGF10*	*H3F3A*	*IRS1*	*MGA*	*PARK2*	*PTPN11*	*SHQ1*	*TMPRSS2*
ARID1A	*CD276*	*E2F3*	*FGF14*	*H3F3B*	*IRS2*	*MITF*	*PARP1*	*PTPRD*	*SLIT2*	*TNFAIP3*
ARID1B	*CD74*	*EED*	*FGF19*	*H3F3C*	*JAK1*	*MLH1*	*PAX3*	*PTPRS*	*SLX4*	*TNFRSF14*
ARID2	*CD79A*	*EGFL7*	*FGF2*	*HGF*	*JAK2*	*MLL*	*PAX5*	*PTPRT*	*SMAD2*	*TOP1*
ARID5B	*CD79B*	*EGFR*	*FGF23*	*HIST1H1C*	*JAK3*	*MLLT3*	*PAX7*	*QKI*	*SMAD3*	*TOP2A*
ASXL1	*CDC73*	*EIF1AX*	*FGF3*	*HIST1H2BD*	*JUN*	*MPL*	*PAX8*	*RAB35*	*SMAD4*	*TP53*
ASXL2	*CDH1*	*EIF4A2*	*FGF4*	*HIST1H3A*	*KAT6A*	*MRE11A*	*PBRM1*	*RAC1*	*SMARCA4*	*TP63*
ATM	*CDK12*	*EIF4E*	*FGF5*	*HIST1H3B*	*KDM5A*	*MSH2*	*PDCD1*	*RAD21*	*SMARCB1*	*TRAF2*
ATR	*CDK4*	*EML4*	*FGF6*	*HIST1H3C*	*KDM5C*	*MSH3*	*PDCD1LG2*	*RAD50*	*SMARCD1*	*TRAF7*
ATRX	*CDK6*	*EP300*	*FGF7*	*HIST1H3D*	*KDM6A*	*MSH6*	*PDGFRA*	*RAD51*	*SMC1A*	*TSC1*
AURKA	*CDK8*	*EPCAM*	*FGF8*	*HIST1H3E*	*KDR*	*MST1*	*PDGFRB*	*RAD51B*	*SMC3*	*TSC2*
AURKB	*CDKN1A*	*EPHA3*	*FGF9*	*HIST1H3F*	*KEAP1*	*MST1R*	*PDK1*	*RAD51C*	*SMO*	*TSHR*
AXIN1	*CDKN1B*	*EPHA5*	*FGFR1*	*HIST1H3G*	*KEL*	*MTOR*	*PDPK1*	*RAD51D*	*SNCAIP*	*U2AF1*
AXIN2	*CDKN2A*	*EPHA7*	*FGFR2*	*HIST1H3H*	*KIF5B*	*MUTYH*	*PGR*	*RAD52*	*SOCS1*	*VEGFA*
AXL	*CDKN2B*	*EPHB1*	*FGFR3*	*HIST1H3I*	*KIT*	*MYB*	*PHF6*	*RAD54L*	*SOX10*	*VHL*
B2M	*CDKN2C*	*ERBB2*	*FGFR4*	*HIST1H3J*	*KLF4*	*MYC*	*PHOX2B*	*RAF1*	*SOX17*	*VTCN1*
BAP1	*CEBPA*	*ERBB3*	*FH*	*HIST2H3A*	*KLHL6*	*MYCL1*	*PIK3C2B*	*RANBP2*	*SOX2*	*WISP3*
BARD1	*CENPA*	*ERBB4*	*FLCN*	*HIST2H3C*	*KMT2B*	*MYCN*	*PIK3C2G*	*RARA*	*SOX9*	*WT1*
BBC3	*CHD2*	*ERCC1*	*FLI1*	*HIST2H3D*	*KMT2C*	*MYD88*	*PIK3C3*	*RASA1*	*SPEN*	*XIAP*
BCL10	*CHD4*	*ERCC2*	*FLT1*	*HIST3H3*	*KMT2D*	*MYOD1*	*PIK3CA*	*RB1*	*SPOP*	*XPO1*
BCL2	*CHEK1*	*ERCC3*	*FLT3*	*HLA-A*	*KRAS*	*NAB2*	*PIK3CB*	*RBM10*	*SPTA1*	*XRCC2*
BCL2L1	*CHEK2*	*ERCC4*	*FLT4*	*HLA-B*	*LAMP1*	*NBN*	*PIK3CD*	*RECQL4*	*SRC*	*YAP1*
BCL2L11	*CIC*	*ERCC5*	*FOXA1*	*HLA-C*	*LATS1*	*NCOA3*	*PIK3CG*	*REL*	*SRSF2*	*YES1*
BCL2L2	*CREBBP*	*ERG*	*FOXL2*	*HNF1A*	*LATS2*	*NCOR1*	*PIK3R1*	*RET*	*STAG1*	*ZBTB2*
BCL6	*CRKL*	*ERRFI1*	*FOXO1*	*HNRNPK*	*LMO1*	*NEGR1*	*PIK3R2*	*RFWD2*	*STAG2*	*ZBTB7A*
BCOR	*CRLF2*	*ESR1*	*FOXP1*	*HOXB13*	*LRP1B*	*NF1*	*PIK3R3*	*RHEB*	*STAT3*	*ZFHX3*
BCORL1	*CSF1R*	*ETS1*	*FRS2*	*HRAS*	*LYN*	*NF2*	*PIM1*	*RHOA*	*STAT4*	*ZNF217*
BCR	*CSF3R*	*ETV1*	*FUBP1*	*HSD3B1*	*LZTR1*	*NFE2L2*	*PLCG2*	*RICTOR*	*STAT5A*	*ZNF703*
BIRC3	*CSNK1A1*	*ETV4*	*FYN*	*HSP90AA1*	*MAGI2*	*NFKBIA*	*PLK2*	*RIT1*	*STAT5B*	*ZRSR2*
BLM	*CTCF*	*ETV5*	*GABRA6*	*ICOSLG*	*MALT1*	*NKX2-1*	*PMAIP1*	*RNF43*	*STK11*	
BMPR1A	*CTLA4*	*ETV6*	*GATA1*	*ID3*	*MAP2K1*	*NKX3-1*	*PMS1*	*ROS1*	*STK40*	
BRAF	*CTNNA1*	*EWSR1*	*GATA2*	*IDH1*	*MAP2K2*	*NOTCH1*	*PMS2*	*RPS6KA4*	*SUFU*	
BRCA1	*CTNNB1*	*EZH2*	*GATA3*	*IDH2*	*MAP2K4*	*NOTCH2*	*PNRC1*	*RPS6KB1*	*SUZ12*	
BRCA2	*CUL3*	*FAM123B*	*GATA4*	*IFNGR1*	*MAP3K1*	*NOTCH3*	*POLD1*	*RPS6KB2*	*SYK*	
					RNA content*					
ABL1	*BCL2*	*CSF1R*	*ESR1*	*EWSR1*	*FLI1*	*KIF5B*	*MSH2*	*NRG1*	*PAX7*	*RAF1*
AKT3	*BRAF*	*EGFR*	*ETS1*	*FGFR1*	*FLT1*	*KIT*	*MYC*	*NTRK1*	*PDGFRA*	*RET*
ALK	*BRCA1*	*EML4*	*ETV1*	*FGFR2*	*FLT3*	*MET*	*NOTCH1*	*NTRK2*	*PDGFRB*	*ROS1*
AR	*BRCA2*	*ERBB2*	*ETV4*	*FGFR3*	*JAK2*	*MLL*	*NOTCH2*	*NTRK3*	*PIK3CA*	*RPS6KB1*
AXL	*CDK4*	*ERG*	*ETV5*	*FGFR4*	*KDR*	*MLLT3*	*NOTCH3*	*PAX3*	*PPARG*	*TMPRSS2*

Table S1. Overview of tumor sample characteristics

	M0 (N = 20)	LM (N = 10)	PM (N = 9)	*p* value
**Tumor cell percentage** (%) – *median (Q1 – Q3)*	75.00<br>(62.50 – 78.75)	55.00<br>(37.50 – 70.00)	70.00<br>(50.00 – 75.00)	**0.011**[a*]
**DNA fragmentation** (bp) – *median (Q1 – Q3)*	300<br>(300 - 400)	300<br>(300 – 400)	300<br>(300 - 300)	0.216[a]
**RNA fragmentation** (DV200) – *median (Q1 – Q3)*	*n = 10*<br>45.44<br>(53.16 – 56.16)	*n = 10*<br>54.07<br>(41.94 – 62.48)	*n = 9*<br>54.95<br>(34.23 – 64.75)	0.462[a]

[a]Kruskall-Wallis Test; *pairwise comparison showed a significant difference (*p* = 0.002) between LM and M0 group, which remained significant after Bonferroni correction (*p* = 0.006).
M0, no metastases; LM, liver metastases; PM, peritoneal metastases; bp, base pairs; Q1 – Q3, quartile 1 – quartile 3

Table S2.
(1) Overview of DNA sequencing (likely) pathogenic variant types and amplifications of total cohort

Pathogenic variant type*	Total freq.	M0 (N = 20)		LM (N = 9)		PM (N = 9)		Total pat.	*p* value[a]
		#	*n (%)*	*#*	*n (%)*	*#*	*n (%)*		
**Missense**	142	74	20 (100)	23	8 (89)	45	9 (100)	37	0.474
**Frameshift**	114	91	14 (70)	9	5 (56)	14	6 (67)	25	0.899
**Nonsense**	71	32	18 (90)	15	8 (89)	24	8 (89)	34	1.000
**InFrameDeletion**	6	5	5 (25)	0	-	1	1 (11)	6	0.271
**SpliceAcceptorSNV**	7	2	2 (10)	1	1 (11)	4	1 (11)	4	1.000
**SpliceDonorSNV**	6	3	3 (15)	0	-	3	2 (22)	5	0.397
**IntronicDeletion**	4	4	3 (15)	0	-	0	-	3	0.403
**InFrameInsertion**	2	0	-	2	2 (22)	0	-	2	0.102
**SpliceDonorInsertion**	1	1	1 (5)	0	-	0	-	1	1.000
**CdsStartSNV**	1	0	-	0	-	1	1 (11)	1	0.474
**Amplification****	11	2	2 (10)	7	3 (33)	2	1 (11)	6	0.375

[a]Fisher-Freeman-Halton Exact Test**;** *Allele frequency ≥ 5%; ** Fold change ≥ 3 Pat., patients; freq., frequency

(2) Overview of DNA sequencing (likely) pathogenic variant types and amplifications after MSI samples exclusion

Pathogenic variant type*	Total freq.	M0 (N = 16)		LM (N = 9)		PM (N = 8)		Total pat.	*p* value[a]
		#	*n (%)*	*#*	*n (%)*	*#*	*n (%)*		
**Missense**	75	34	16 (100)	23	8 (89)	18	8 (100)	32	0.515
**Frameshift**	35	14	11 (69)	9	6 (67)	12	6 (75)	23	1.000
**Nonsense**	44	22	14 (88)	15	9 (100)	7	7 (88)	30	0.591
**InFrameDeletion**	4	3	3 (19)	0	-	1	1 (13)	4	0.533
**SpliceAcceptorSNV**	2	1	1 (6)	1	1 (11)	0	-	2	1.000
**SpliceDonorSNV**	2	1	1 (6)	0	-	1	1 (13)	2	0.727
**InFrameInsertion**	2	0	-	2	2 (22)	0	-	2	0.121
**Amplification****	11	2	2 (13)	7	3 (33)	2	1 (13)	6	0.499

[a]Fisher-Freeman-Halton Exact Test**;** *Allele frequency ≥ 5%; ** Fold change ≥ 3; Pat., patients; freq., frequency

Table S3. Detailed overview of (likely) pathogenic DNA mutations and amplifications

Patient (M group)	Gene	cDNA	Protein	Allele frequency	Exon	Consequence
**1 (M0)**	APC	c.4135G>T	p.E1379*	54%	2	Nonsense
	TP53	c.817C>T	p.R273C	40%	8	Missense
**2 (M0)**	APC	c.2910delT<br>c.4057G>T	p.S970Rfs*10<br>p.E1353*	29%<br>13%	16<br>16	Frameshift<br>Nonsense
	RAC1	c.608_610delAGA	p.K203delK	15%	7	InFrameDeletion
	GATA4	c.848G>A	p.R283H	13%	4	Missense
	NF1	c.3805_3808dupGACT	p.S1270*fs*1	5%	28	Nonsense
	CUL3	c.944A>T	p.E315V	5%	7	Missense
**3 (M0)**	APC	c.4012C>T	p.Q1338*	43%	16	Nonsense
	NRAS	c.181C>A	p.Q61K	26%	3	Missense
	AR	c.1174C>T	p.P392S	100%	1	Missense
	ATM	c.2921+1G>A	.	30%	-	SpliceDonorSNV
	PARK2	c.736A>C	p.S246R	24%	7	Missense
	CREBBP	c.1289G>A	p.C430Y	18%	5	Missense
	ATM	c.9150delA	p.G3051Dfs*24	17%	63	Frameshift
	B2M	c.89A>G	p.Y30C	12%	2	Missenese
**4 (M0)**	APC	c.2635C>T	p.Q879*	34%	16	Nonsense
	KRAS	c.35G>T	p.G12V	23%	2	Missense
	TP53	c.586C>T	p.R196*	30%	6	Nonsense
	GATA3	c.1163A>G	p.K388R	45%	6	Missense
	PRKDC	c.10412_10416delTAGAA<br>c.10404_10405delTC	p.I3471Tfs*50<br>p.Q3469Dfs*53	4%<br>5%	74	Frameshift
**5 (M0)**	APC	c.2545_2546delGA<br>c.4240delG	p.D849*fs*1<br>p.V1414*fs*1	23%	16	Frameshift<br>Frameshift
	TP53	c.844C>T	p.R282W	29%	8	Missense
	TGFBR1	c.643C>T	p.R215*	25%	4	Nonsense

	APC	c.4285C>T	p.Q1429*	27%	16	Nonsense
	KRAS	c.38_39delinsAT	p.G13D	37%	2	Missense
	HRAS	c.187_189delGAG	p.E63delE	25%	3	InFrameDeletion
**7 (M0)**	APC	c.4280delC	p.P1427Lfs*46	36%	16	Frameshift
	TP53	c.586C>T<br>c.743G>A	p.R196*<br>p.R248Q	2%<br>52%	6<br>7	Nonsense<br>Missense
	SMAD4	c.1082G>A	p.R361H	30%	9	Missense
	FLI1	c.493A>C	p.K165Q	76%	4	Missense
	SOX9	c.1231C>T	p.Q411*	25%	3	Nonsense
**8 (M0)**	APC	c.3910delA<br>c.904C>T	p.I1304*fs*1<br>p.R302*	17%<br>31%	16<br>9	Frameshift<br>Nonsense
	TP53	c.476C>T	p.A159V	43%	5	Missense
	SMARCB1	c.1121G>A	p.R374Q	42%	9	Missense
	CDK8	c.103G>A	p.V35I	34%	1	Missense
**9 (M0)**	APC	c.3925G>T	p.E1309*	66%	16	Nonesense
	TP53	c.6735_676dupCCTAGGTTG<br>c.673-1G>T	p.G226Afs*2<br>-	19%<br>36%	7<br>-	Frameshift<br>SpliceAcceptorSNV
	PIK3C2B	c.4382_4383insT	p.E1463*fs*1	47%	30	Nonsense
	MUTYH	c.536A>G	p.Y179C	17%	7	Missense
	PAK3	c.807delG	p.L269Ffs*14	5%	8	Framshift
	NRAS	.	.	.	.	Amplification
**10 (M0)**	APC	c.3964G>T	p.E1322*	75%	16	Nonsense
	BRAF	c.1024delA	p.I342Ffs*30	5%	8	Frameshift
	TP53	c.818G>A	p.R273H	73%	8	Missense
	POLD1	c.2954G>C	p.R985P	18%	24	Missense
	BARD1	c.1811-3dupT	.	6%	-	SpliceAcceptorInsertion
	FAT1	c.5916delC	p.S1972Rfs*6	5%	10	Frameshift
	APC	c.3907C>T	p.Q1303*	56%	16	Nonsense

**11**	TP53	c.641A>G	p.H214R	51%	6	Missense
**(M0)**	SMAD3	c.733G>A	p.G245R	58%	6	Missense
**12**	BRAF	c.1799T>A	p.V600E	10%	15	Missense
**(M0)**	PIK3CA	c.3128T>C	p.M1043T	10%	21	Missense
	PIM1	c.83G>T	p.G28V	48%	2	Missense
**MSI**	ESR1	c.805C>T	p.R269C	44%	16	Missense
**TMB**	RNF43	c.1976delG	p.G659Vfs*41	22%	8	Frameshift
	PTEN	c.649G>T	p.V217F	14%	7	Missense
	MRE11A	c.1757C>A	p.S586*	14%	15	Nonsense
	ZNF217	c.724delA	p.T242Lfs*30	13%	2	Frameshift
	PAK3	c.539-1G>T	.	12%	-	SpliceAcceptorSNV
	ARID1A	c.1636delC	p.Q546Rfs*73	10%	3	Frameshift
	CIC	c.3347dupC	p.S1117Kfs*34	10%	14	Frameshift
	HNF1A	c.864delG	p.P291Qfs*51	10%	4	Frameshift
	MGA	c.1246_1249delAACA	p.N416Vfs*7	10%	3	Frameshift
	CHD4	c.3283C>T	p.R1095C	9%	22	Missense
	ZBTB2	c.484C>T	p.Q162*	9%	3	Nonsense
	GNAS	c.349G>A	p.V117M	8%	5	Missense
	FLI1	c.490G>C	p.G164R	8%	4	Missense
	POLE	c.3510dupA	p.L1171Tfs*6	7%	29	Frameshift
	JAK1	c.1645C>T	p.R549*	7%	11	Nonsense
**29**	PIK3CA	c.3062A>G	p.Y1021C	19%	21	Missense
**(M0)**		c.3139C>T	p.H1047Y	16%		Missense
	BRAF	c.1799T>A	p.V600E	20%	15	Missense
**MSI**	FAM175A	c.796+1G>A	.	54%	-	SpliceDonorSNV
**TMB**	BARD1	c.1921C>T	p.R641*	27%	10	Nonsense
	ABL1	c.929C>T	p.P310L	25%	6	Missense
	XRCC2	c.94_97delTTTG	p.F32Lfs*29	22%	2	Frameshift
	TET2	c.436_437delGA	p.D146*fs*1	21%	3	Frameshift
	FLT3	c.2955_2956delTC	p.P986Afs*164	21%	24	Frameshift
	PAX7	c.906delC	p.Y303Tfs*64	21%	6	Frameshift
	ATM	c.8432dupA	p.S2812Vfs*3	21%	58	Frameshift
	ZRSR2	c.938G>T	p.G313V	20%	11	Missense
	MGA	c.4357delT	p.Y1453Mfs*27	20%	13	Frameshift

	DNMT3A	c.2503A>G	p.T835A	20%	22	Missense
	MGA	c.4357delT	p.Y1453Mfs*27	20%	13	Frameshift
	EML4	c.387delA	p.E130Rfs*43	19%	4	Frameshift
	FAT1	c.4603C>T	p.R1535*	19%	9	Nonsense
	BLM	c.3210+9dupA	.	18%	-	SpliceDonorInsertion
	BRCA2	c.9253delA	p.T3085Qfs*19	18%	24	Frameshift
	CDKN2A	c.85C>T	p.R29W	18%	1	Missense
	SOX9	c.1121dupC	p.Q375Afs*203	17%	3	Frameshift
	CSF3R	c.1404delC	p.S469Afs*22	17%	11	Frameshift
	IRS1	c.2456delG	p.G819Dfs*124	17%	1	Frameshift
	MAGI2	c.2846-3delT	.	17%	-	IntronicDeletion
	SOX9	c.1121dupC	p.Q375Afs*203	17%	3	Frameshift
	CSF3R	c.1404delC	p.S469Afs*22	17%	11	Frameshift
	ERBB3	c.694G>A	p.A232T	17%	6	Missense
	FOXL2	c.576delC	p.K193Sfs*78	16%	1	Frameshift
	GATA2	c.818dupG	p.P274Tfs*8	16%	3	Frameshift
	SETBP1	c.1261delA	p.R421Dfs*23	16%	4	Frameshift
	ALK	c.2633G>A	p.G878D	15%	16	Missense
	BCORL1	c.1929delC	p.M644Cfs*4	13%	3	Frameshift
	AXIN2	c.1994dupG	p.N666Qfs*41	13%	8	Frameshift
	ALOX12B	c.1303G>A	p.V435I	12%	10	Missense
	TGFBR1	c.557C>T	p.T186M	9%	3	Missense
	CARD11	c.1663delC	p.R555Gfs*45	8%	3	Frameshift
	CASP8	c.1173delC	p.I392Sfs*4	8%	8	Frameshift
	ATRX	c.6111-3delT	.	7%	-	IntronicDeletion
	NOTCH4	c.529delC	p.Q177Rfs*75	6%	4	Frameshift
	FAM175A	c.796+1G>A	.	54%	-	SpliceDonorSNV
	RNF43	c.1976delG	p.G659Vfs*41	9%	8	Frameshift
		c.354delC	p.C119Afs*39	20%	2	Frameshift
		c.349dupC	p.R117Pfs*8	16%	2	Frameshift
**14 (M0)**	BARD1	c.1921C>T	p.R641*	27%	10	Nonsense
	PIK3CA	c.3062A>G	p.Y1021C	19%	21	Missense
		c.3139C>T	p.H1047Y	16%		Missense
	BRAF	c.1799T>A	p.V600E	20%	15	Missense

	ATRX	c.6111-3delT	.	7%	-	IntronicDeletion
	MAGI2	c.2846-3delT	.	17%	-	IntronicDeletion
	RBM10	c.534delT	p.F178Lfs*88	38%	6	Frameshift
	KRAS	c.40G>A	p.V14I	21%	2	Missense
	TP53	c.383dupC	p.A129Cfs*20	24%	5	Frameshift
	ALK	c.523_540delAGTTGGTGGATTCGCCAA	p.S175_Q180delSWWIRQ	43%	1	InFrameDeletion
	PBRM1	c.2566C>T	p.R856W	23%	17	Missense
	AKT3	c.740G>A	p.R247H	19%	9	Missense
	JUN	c.930_931delGA	p.K311Sfs*6	17%	1	Frameshift
	AXIN2	c.1228dupA	p.T410Nfs*52	16%	6	Frameshift
	FGFR1	.	.	.	.	Amplification
**16 (M0)**<br><br>**MSI TMB**	BRAF	c.1799T>A	p.V600E	25%	15	Missense
	PIK3CA	c.328_330delGAA	p.E110delE	21%	2	InFrameDeletion
	TP53	c.455delC	p.P152Rfs*18	38%	5	Frameshift
	DDR2	c.1552delG	p.V518Cfs*9	46%	14	Frameshift
	FOXL2	c.18delC	p.E7Sfs*143	39%	1	Frameshift
	CDKN2A	c.202G>A	p.A68T	37%	2	Missense
	MEN1	c.1366G>T	p.V456L	34%	10	Missense
	CREBBP	c.1816C>T	p.H606Y	33%	8	Missense
	FANCE	c.967C>T	p.Q323*	32%	4	Nonsense
	KDM5C	c.1354G>A	p.G452S	32%	10	Missense
	BCR	c.2115+2T>C	.	32%	-	SpliceDonorSNV
	BCOR	c.2428C>T	p.R810*	31%	4	Nonsense
	LRP1B	c.11869dupA	p.R3957Kfs*11	31%	77	Frameshift
	CUL3	c.2231G>A	p.R744H	30%	16	Missense
	JAK2	c.544G>T	p.A182S	30%	6	Missense
	IRF2	c.86A>C	p.K29T	29%	2	Missense
	MSH6	c.3163G>A	p.A1055T	29%	4	Missense
	RARA	c.89delC	p.P30Lfs*12	29%	2	Frameshift
	ZBTB7A	c.522delC	p.A175Rfs*43	29%	2	Frameshift
	GNAS	c.1066C>T	p.R356C	28%	13	Missense
	EED	c.1124A>C	p.K375T	28%	10	Missense
	TSHR	c.16_17dupTT	p.L6Ffs*53	28%	2	Frameshift

BRCA2	c.36delT	p.F12Lfs*13	27%	2	Frameshift
IL7R	c.799delA	p.R267Gfs*28	27%	6	Frameshift
KDM5A	c.23delG	p.G8Afs*58	27%	1	Frameshift
ATRX	c.6111-3delT	.	27%	-	IntronicDeletion
POLD1	c.342delG	p.P116Hfs*53	26%	4	Frameshift
HNRNPK	c.746G>A	p.R249H	26%	11	Missense
RPTOR	c.3010C>T	p.Q1004*	26%	25	Nonsense
ETV6	c.1226A>T	p.K409M	25%	7	Missense
FGFR3	c.1206dupC	p.K403Qfs*93	25%	9	Frameshift
SOCS1	c.150delC	p.G51Afs*34	24%	2	Frameshift
SOX9	c.66delC	p.S23Afs*38	24%	1	Frameshift
GLI1	c.821delG	p.G274Afs*6	23%	8	Frameshift
ICOSLG	c.718_719delGA	p.D240Qfs*103	23%	5	Frameshift
IRF4	c.623delC	p.P208Qfs*9	23%	5	Frameshift
EPHA3	c.2538delC	p.M847Wfs*10	22%	15	Frameshift
MAP2K1	c.320T>C	p.I107T	22%	3	Missense
KIF5B	c.1052dupA	p.N351Kfs*2	21%	11	Frameshift
TOP2A	c.3613delA	p.T1205Hfs*19	21%	28	Frameshift
SPTA1	c.4866G>A	p.W1622*	19%	34	Nonsense
AXIN1	c.1034delC	p.P345Hfs*69	31%	4	Frameshift
	c.792delC	p.G265Efs*149	26%	2	Frameshif
AXIN2	c.2011delC	p.R671Afs*18	8%	8	Frameshift
	c.1994delG	p.G665Afs*24	14%		Frameshift
BCORL1	c.220delG	p.A74Qfs*42	28%	3	Frameshift
	c.5041_5042delCC	.P1681Rfs*4	26%	12	Frameshift
NOTCH4	c.4322delC	p.P1441Lfs*17	27%	24	Frameshift
	c.2125delC	p.Q709Sfs*22	23%	13	Frameshift
RASA1	c.625_626delAG	p.S209*fs*1	26%	2	Frameshift
	c.2513delA	p.N838Mfs*4	24%	19	Frameshift
RNF43	c.1976delG	p.G659Vfs*41	28%	8	Frameshift
	c.468delG	p.L157*fs*1	30%	4	Frameshift
PAX5	c.295delA	p.I99Sfs*60	21%	3	Frameshift
	c.81delT	p.F27Lfs*2	21%	2	Frameshift

	JAK1	c.2580delA	p.K860Nfs*16	25%	19	Frameshift
		c.1289dupC	p.L431Vfs*22	30%	9	Frameshift
		c.425delA	p.K142Rfs*26	24%	5	Frameshift
**17**	TP53	c.574C>T	p.Q192*	55%	6	Nonsense
**(M0)**	CTNNB1	c.121A>G	p.T41A	52%	3	Missense
**26**	APC	c.3807_3808delAT	p.I1269Mfs*6	26%	16	Frameshift
**(M0)**	TP53	c.817C>T	p.R273C	17%	8	Missense
**19**	KRAS	c.35G>T	p.G12V	26%	2	Missense
**(M0)**	BRAF	c.1406G>C	p.G469A	22%	11	Missense
	PIK3CA	c.335T>A	p.I112N	12%	2	Missense
**MSI**	RNF43	c.1976delG	p.G659Vfs*41	51%	8	Frameshift
**TMB**	ATRX	c.6111-3delT	.	47%	-	IntronicDeletion
	GATA1	c.842C>T	p.A281V	45%	5	Missense
	PARK2	c.1298A>C	p.H433P	41%	12	Missense
	RAD54L	c.304delA	p.R102Gfs*25	27%	5	Frameshift
	CTCF	c.1928delC	p.P643Qfs*43	26%	11	Frameshift
	TGFBR2	c.1157T>C	p.L386P	26%	5	Missense
	PRKCI	c.665C>G	p.S222*	25%	8	Nonsense
	PREX2	c.141G>A	p.S47S	25%	1	Synonymous
	CDKN1B	c.214_215dupGG	p.K73Afs*47	24%	1	Frameshift
	NBN	c.247dupA	p.M83Nfs*23	24%	3	Frameshift
	MAGI2	c.104delG	p.G35Afs*13	24%	1	Frameshift
	MLL	c.2318delC	p.P773Rfs*8	24%	3	Frameshift
	MUTYH	c.596delG	p.G199Afs*41	23%	8	Frameshift
	IDH2	c.202_204delGAG	p.E68delE	22%	2	InFrameDeletion
		c.1620delA	p.K540Nfs*32	22%	17	Frameshift
	MSH3	c.2582delA	p.N861Mfs*6	19%	19	Frameshift
	SETD2	c.843dupA	p.E282Rfs*9	17%	3	Frameshift
**23**	APC	c.3253A>T	p.K1085*	26%	16	Nonsense
**(M0)**		c.4585C>T	p.Q1529*	19%		Nonsense
	KRAS	c.35G>T	p.G12V	33%	2	Missense
	SMAD4	c.1256G>T	p.G419V	40%	10	Missense
	TP53	c.722C>A	p.S241Y	38%	7	Missense

	FAM123B	c.1834G>T	p.E612*	34%	2	Nonsense
	IRS1	c.2512C>T	p.Q838*	18%	1	Nonsense
**21 (LM)**	APC	c.3860_3879dupTAGGATGTAATCAGACGACA	p.Q1294*fs*1	21%	16	Nonsense
	TP53	c.844_849dupCGGCGC	p.R282_R283dupRR	29%	8	InFrameInsertion
**22 (LM)**	APC	c.4254_4269delAAGCCCCAGTGATCTT	p.S1419Qfs*49	51%	16	Frameshift
	NRAS	c.182A>T	p.Q61L	55%	3	Missense
	TP53	c.817C>T	p.R273C	36%	8	Missense
	SMAD4	c.1067C>A	p.P356H	41%	9	Missense
	SOX9	c.1319dupA	p.Y440*fs*1	55%	3	Nonsense
	PAX8	c.275T>C	p.I92T	50%	4	Missense
	GPS2	c.139G>T	p.E47*	35%	3	Nonsense
	RAF1	c.770C>T	p.S257L	28%	7	Missense
**13 (LM)**	APC	c.3871C>T	p.Q1291*	78%	16	Nonsense
	SMAD4	c.170_171delTA	p.L57Yfs*12	10%	2	Frameshift
	TP53	c.524G>A	p.R175H	80%	5	Missense
	KRAS	c.35G>C	p.G12A	79%	2	Missense
	BRCA2	.	.	.	.	Amplification
	LAMP1	.	.	.	.	Amplification
**20 (LM)**	APC	c.3927_3931delAAAGA<br>c.2413C>T	p.E1309Dfs*4<br>p.R805*	8%<br>36%	16<br>16	Frameshift<br>Nonsense
	TP53	c.493C>T	p.Q165*	51%	5	Nonsense
	PIK3R1	c.1389_1390insATT	p.Y463_D464insI	39%	11	InFrameInsertion
	H3F3A	c.350G>A	p.R117H	36%	3	Missense
	PBRM1	c.3463G>T	p.E1155*	22%	23	Nonsense
	SOX9	c.437T>A<br>c.611dupT	p.L146Q<br>p.K205Qfs*47	26%<br>34%	2<br>2	Missense<br>Frameshift
**24 (LM)**	APC	c.3294T>A<br>c.3871C>T	p.C1098*<br>p.Q1291*	15%<br>16%	16	Nonsense<br>Nonsense
	KRAS	c.35G>A	p.G12D	25%	2	Missense
	PIK3CA	c.3127A>G<br>c.344G>T	p.M1043V<br>p.R115L	11%<br>10%	21<br>2	Missense<br>Missense

	SMAD4	c.1577A>C	p.E526A	25%	12	Missense
	MED12	c.97G>C	p.E33Q	48%	1	Missense
	PAK3	c.1201G>T	p.A401S	12%	12	Missense
	SPTA1	c.3136C>T	p.R1046*	15%	22	Nonsense
	ATRX	c.4318-2A>G	.	8%	-	SpliceAcceptorSNV
	NF1	c.5050delT	p.S1684Pfs*14	2%	37	Frameshift
**25 (LM)**	APC	c.4222G>T	p.E1408*	21%	16	Nonsense
	TP53	c.916C>T	p.R306*	17%	8	Nonsense
	KRAS	c.35G>A	p.G12D	23%	2	Missense
	CCND1	.	.	.	.	Amplification
	FGF3	.	.	.	.	Amplification
**27 (LM)**	APC	c.637C>T	p.R213*	57%	6	Nonsense
	TP53	c.817C>T	p.R273C	61%	8	Missense
	RAD50	c.1232_1242delCCAACCAACTG	p.A411Dfs*3	17%	8	Frameshift
	MLH1	c.1964T>C	p.I655T	55%	17	Missense
	CDKN2A	c.301G>T	p.G101W	51%	2	Missense
	FGF23	.	.	.	.	Amplification
	FGF6	.	.	.	.	Amplification
	KRAS	.	.	.	.	Amplification
**28 (LM)**	APC	c.2825dupA	p.N942Kfs*4	32%	16	Frameshift
		c.4216C>T	p.Q1406*	35%	16	Nonsense
	KRAS	c.34G>T	p.G12C	34%	2	Missense
	BCORL1	c.2350C>T	p.R784*	79%	3	Nonsense
	SOX9	c.1113_1117dupGGCAC	p.P373Rfs*12	34%	3	Frameshift
	PARK2	c.476C>T	p.P159L	32%	4	Missense
	MITF	c.836G>A	p.R279Q	18%	8	Missense
	NOTCH1	c.7541_7542delCT	p.P2514Rfs*4	19%	34	Frameshift
	FAT1	c.3744_3754delGTTCTACAAAA	p.K1248Nfs*5	5%	5	Frameshift
**30 (LM)**	FAM123B	c.994delA	p.I332*fs*1	77%	2	Frameshift
	ATM	c.9022C>T	p.R3008C	36%	63	Missense
	APC	c.3927_3931delAAAGA	p.E1309Dfs*4	17%	16	Frameshift

**31 (PM)**		c.1495C>T	p.R499*	23%	12	Nonsense
	ATM	c.5216dupA	p.N1739Kfs*10	34%	35	Frameshift
	PAX3	c.290G>A	p.R97H	20%	2	Missense
	MDM4	.	.	.	.	Amplification
	ERBB2	.	.	.	.	Amplification
**32 (PM)**	BRAF	c.1799T>A	p.V600E	39%	15	Missense
	TP53	c.637C>T	p.R213*	56%	6	Nonsense
	SOX2	c.804_805delinsAC	p.D269H	42%	1	Missense
	RHEB	c.275+2T>G	.	22%	-	SpliceDonorSNV
	INSR	c.3560C>T	p.T1187M	16%	20	Missense
**33 (PM)**	APC	c.3934G>T	p.G1312*	56%	16	Nonsense
	KRAS	c.35G>A	p.G12D	46%	2	Missense
	SMAD4	c.1201dupT	p.C401Lfs*3	35%	10	Frameshift
	TP53	c.383delC	p.P128Lfs*42	40%	5	Frameshift
**34 (PM)**	APC	c.4214_4247del TTCAGAGTGAACCATGCAGTGGAATGGTAAGTGG	p.V1405Afs*3	18%	16	Frameshift
	TP53	c.224dupC	p.A76Cfs*73	29%	4	Frameshift
	FLCN	c.616A>G	p.K206E	32%	6	Missense
	TCF7L2	c.268C>T	p.Q90*	27%	3	Nonsense
**35 (PM)**	APC	c.3778C>T<br>c.4654G>T	p.Q1260*<br>p.E1552*	26%<br>22%	16	Nonsense<br>Nonsense
	KRAS	c.175G>A	p.A59T	17%	3	Missense
**TMB**	NRAS	c.510dupA	p.L171Tfs*5	18%	5	Frameshift
**MSI**	PIK3CA	c.929G>A	p.R310H	10%	5	Missense
	ERCC5	c.2974C>T	p.R992*	48%	15	Nonsense
	MSH6	c.3772C>T	p.Q1258*	44%	8	Nonsense
	FAM123B	c.1756C>T	p.R586*	39%	2	Nonsense
	H3F3A	c.350G>A	p.R117H	33%	3	Missense
	GNAQ	c.477-2A>G	.	29%	-	SpliceAcceptorSNV
	KAT6A	c.2323C>T	p.R775C	28%	15	Missense
	ERBB3	c.695C>T	p.A232V	27%	6	Missense
	FLT3	c.2480T>C	p.I827T	26%	20	Missense

LRP1B	c.9032C>T	p.S3011L	26%	56	Missense
BRD4	c.3223C>T	p.Q1075*	26%	15	Nonsense
PHOX2B	c.323C>T	p.A108V	26%	2	Missense
EGFR	c.2135T>C	p.F712S	25%	18	Missense
GNA11	c.656G>A	p.C219Y	24%	5	Missense
TRAF7	c.91G>A	p.E31K	24%	3	Missense
HRAS	c.291-1G>T	.	24%	-	SpliceAcceptorSNV
LATS1	c.2785C>T	p.Q929*	24%	7	Nonsense
PARK2	c.844C>T	p.Q282*	24%	7	Nonsense
WISP3	c.382G>A	p.E128K	24%	2	Missense
ABL1	c.908G>T	p.G303V	23%	6	Missense
CREBBP	c.5470G>A	p.A1824T	23%	31	Missense
IDH1	c.874A>G	p.T292A	23%	8	Missense
SMARCB1	c.1097G>A	p.R366H	23%	8	Missense
IGF1R	c.1101delG	p.N368Ifs*17	23%	4	Frameshift
SYK	c.583C>T	p.R195*	23%	4	Nonsense
ARID1A	c.5503C>T	p.Q1835*	22%	20	Nonsense
FGFR2	c.1547C>T	p.A516V	22%	11	Missense
TGFBR2	c.1658G>A	p.R553H	22%	8	Missense
MAPK3	c.171-1G>A	.	22%	-	SpliceAcceptorSNV
NF2	c.774G>A	p.W258*	21%	8	Nonsense
NOTCH1	c.4721T>C	p.L1574P	21%	26	Missense
PPP6C	c.442C>T	p.R148*	21%	5	Nonsense
BCL6	c.1948C>T	p.R650*	21%	9	Nonsense
GNAS	c.325G>A	p.A109T	20%	5	Missense
MITF	c.836G>A	p.R279Q	20%	8	Missense
SMAD3	c.3G>A	.	20%	1	CdsStartSNV
EED	c.540G>A	p.M180I	20%	5	Missense
RARA	c.436C>T	p.Q146*	20%	4	Nonsense
FLCN	c.1579C>T	p.R527*	19%	14	Nonsense
JAK2	c.1310T>C	p.L437S	19%	10	Missense
TCF7L2	c.1318+1G>A	.	19%	-	SpliceDonorSNV
	c.1391G>T	p.R464M	21%	13	Missense
FLT4	c.3122G>A	p.R1041Q	18%	23	Missense
ASXL2	c.3799C>T	p.Q1267*	13%	13	Nonsense

	PAX3	c.62C>T	p.P21L	13%	1	Missense
	PTPRT	c.2857C>T	p.R953*	11%	20	Nonsense
	ALOX12B	c.1510G>A	p.A504T	9%	11	Missense
	CTNNA1	c.361C>T	p.R121*	5%	4	Nonsense
	PTEN	c.209+2T>C	.	5%	-	SpliceDonorSNV
		c.254-1G>A	.	5%	-	SpliceAcceptorSNV
**36 (PM)**	KRAS	c.34G>A	p.G12S	17%	2	Missense
	PIK3CA	c.1633G>A	p.E545K	13%	10	Missense
	TP53	c.455C>T	p.P152L	15%	5	Missense
	DDX41	c.647dupT	p.S217Ifs*4	47%	8	Frameshift
	MSH6	c.2081_2082delGC	p.C694Sfs*3	9%	4	Frameshift
	BRCA1	c.3862G>T	p.E1288*	8%	10	Nonsense
	DHX15	c.139_140insA	p.G47Efs*3	6%	2	Frameshift
	KIF5B	c.1026_1033delGAAGTATG	p.Y344Rfs*6	5%	11	Frameshift
**37 (PM)**	SMAD4	c.1572G>T	p.W524C	23%	12	Missense
	TP53	c.817C>T	p.R273C	33%	8	Missense
	BRAF	c.1799T>A	p.V600E	34%	15	Missense
**38 (PM)**	APC	c.4393_4394delAG	p.S1465Wfs*3	50%	16	Frameshift
	KRAS	c.35G>T	p.G12V	44%	2	Missense
	FAM123B	c.227delG	p.G76Dfs*24	62%	2	Frameshift
	KDM5C	c.1714_1731delAATCCCAACACCCTCATG	p.N572_M577delNPNTLM	6%	12	InFrameDeletion
	SLX4	c.634C>T	p.R212*	30%	3	Nonsense
**40 (PM)**	TP53	c.404G>T	p.C135F	57%	5	Missense
	MUTYH	c.1187G>A	p.G396D	43%	13	Missense
	PREX2	c.1158T>A	p.Y386*	38%	10	Nonsense
	AR	c.2471A>C	p.N824T	26%	7	Missense
	BRAF	c.1799T>A	p.V600E	25%	15	Missense
	FBXW7	c.943G>C	p.A315P	24%	6	Missense

Table S4. Detailed overview percentage of mutated/amplified gene in each subgroup after MSI sample deletion, with statistical analysis

Gene	M0 (%) N = 16	LM (%) N = 9	PM (%) N = 8	P value[a]
**APC**	87.5	88.89	50.0	0.134
**TP53**	75.0	66.67	75.0	0.886
**BARD1**	6.25	0.0	0.0	1.000
**FAT1**	6.25	0.0	0.0	1.000
**POLD1**	6.25	0.0	0.0	1.000
**SMAD3**	6.25	0.0	0.0	1.000
**BRAF**	0.0	0.0	37.5	0.010
**PIK3CA**	0.0	11.11	12.5	0.258
**FLI1**	6.25	0.0	0.0	1.000
**PAK3**	6.25	11.11	0.0	1.000
**SMAD4**	12.5	33.33	25.0	0.476
**KRAS**	31.25	55.56	37.5	0.588
**IDH2**	6.25	0.0	0.0	1.000
**CSF1R**	6.25	0.0	0.0	1.000
**PIK3R1**	6.25	11.11	0.0	1.000
**RBM10**	6.25	0.0	0.0	1.000
**AXIN2**	6.25	0.0	0.0	1.000
**PBRM1**	6.25	11.11	0.0	1.000
**AKT3**	6.25	0.0	0.0	1.000
**ALK**	6.25	0.0	0.0	1.000
**JUN**	6.25	0.0	0.0	1.000
**BRCA2**	0.0	11.11	0.0	0.515
**CDKN2A**	0.0	11.11	0.0	0.515
**CREBBP**	6.25	0.0	0.0	1.000
**CUL3**	6.25	0.0	0.0	1.000
**KDM5C**	0.0	0.0	12.5	0.242
**SOX9**	6.25	33.33	0.0	0.113
**BCORL1**	0.0	11.11	0.0	0.515
**KIF5B**	0.0	0.0	12.5	0.242
**MSH6**	0.0	0.0	12.5	0.242
**SPTA1**	0.0	11.11	0.0	0.515
**ATRX**	0.0	11.11	0.0	0.515
**CTNNB1**	6.25	0.0	0.0	1.000
**MUTYH**	6.25	0.0	12.5	0.727
**PARK2**	6.25	11.11	0.0	1.000
**PREX2**	0.0	0.0	12.5	0.242
**GATA4**	6.25	0.0	0.0	1.000
**RAC1**	6.25	0.0	0.0	1.000
**H3F3A**	0.0	11.11	0.0	0.515
**GPS2**	0.0	11.11	0.0	0.515
**PAX8**	0.0	11.11	0.0	0.515
**RAF1**	0.0	11.11	0.0	0.515
**NRAS**	12.5	11.11	0.0	0.789
**FAM123B**	6.25	11.11	12.5	1.000
**IRS1**	6.25	0.0	0.0	1.000
**MED12**	0.0	11.11	0.0	0.515
**RAD50**	0.0	11.11	0.0	0.515
**MLH1**	0.0	11.11	0.0	0.515
**MITF**	0.0	11.11	0.0	0.515
**NOTCH1**	0.0	11.11	0.0	0.515

**ATM**	6.25	11.11	12.5	1.000
**TGFBR1**	6.25	0.0	0.0	1.000
**AR**	6.25	0.0	12.5	0.727
**B2M**	6.25	0.0	0.0	1.000
**PAX3**	0.0	0.0	12.5	0.242
**INSR**	0.0	0.0	12.5	0.242
**RHEB**	0.0	0.0	12.5	0.242
**SOX2**	0.0	0.0	12.5	0.242
**FLCN**	0.0	0.0	12.5	0.242
**TCF7L2**	0.0	0.0	12.5	0.242
**SMARCB1**	6.25	0.0	0.0	1.000
**HRAS**	6.25	0.0	0.0	1.000
**BRCA1**	0.0	0.0	12.5	0.242
**DHX15**	0.0	0.0	12.5	0.242
**DDX41**	0.0	0.0	12.5	0.242
**SLX4**	0.0	0.0	12.5	0.242
**GATA3**	6.25	0.0	0.0	1.000
**FBXW7**	0.0	0.0	12.5	0.242
**CDK8**	6.25	0.0	0.0	1.000
**PIK3C2B**	6.25	0.0	0.0	1.000
**LAMP1**	0.0	11.11	0.0	0.515
**FGFR1**	6.25	0.0	0.0	1.000
**CCND1**	0.0	11.11	0.0	0.515
**FGF3**	0.0	11.11	0.0	0.515
**FGF23**	0.0	11.11	0.0	0.515
**FGF6**	0.0	11.11	0.0	0.515
**MDM4**	0.0	0.0	12.5	0.242
**ERBB2**	0.0	0.0	12.5	0.242

[a]Fisher-Freeman-Halton Exact test

Supplementary Section S2: Analysis on total cohort (MSI + MSS samples)

173/523 genes were (likely) pathogenic affected, resulting in 415 variant types (Figure S4). Missense, frameshift, and nonsense mutations were most common. When comparing the occurrence of all variant types and amplifications, no significant differences were found.

Well-known oncogenes related to CRC were investigated in each subgroup (Figure 2A). APC mutations occurred most in LM patients (n = 8/9, 88.89%), while TP53 mutations were equally divided into three groups. BRAF mutations did not occur in LM patients and were most present in PM patients (n = 3/9, 33.33%). PIK3CA and NRAS mutations were almost equally distributed. SMAD4 mutations and KRAS mutations were mostly found in LM groups (n = 3/9, 33.33% and n = 5/9, 55.56% respectively) and to a lesser extent in M0 groups. None of the gene alterations were significant different when comparing all groups.

Although no gene variant was significantly higher in the PM group, we identified 34 genes which were solely affected in the PM group (Figure 2B). In total, 32 mutations and two amplifications occurred in 7/9 patients. The majority of the affected genes (91%) occurred in solely patient, except for three gene alterations (e.g., PAX3, FLCN and TCF7L2) in two PM patients, although none of these variants were on the same exon.

A total of 11 gene variants were solely detected in 6 LM patients (Figure 2C). Mutations in GPS2, PAX8, and RAF1 co-occurred in one patient, mutations in RAD50 and MLH1were found in another patient. The same counts for amplifications of CCND1 and FGF3, and of FGF23 and FGF6. The MED12 mutation and LAMP1 amplification were identified in two different patients.

When taking the results of the LM and PM groups together, H3F3A, MITF, and NOTCH1 were mutated in one LM and one PM sample (Figure 2D). The latter occurred in the same PM patient, whilst two patients were involved in the LM group. Both H3F3A and MITF mutations were missense mutations, on the exact same location (exon 3 and 8 respectively). For NOTCH1 different variant types were involved.

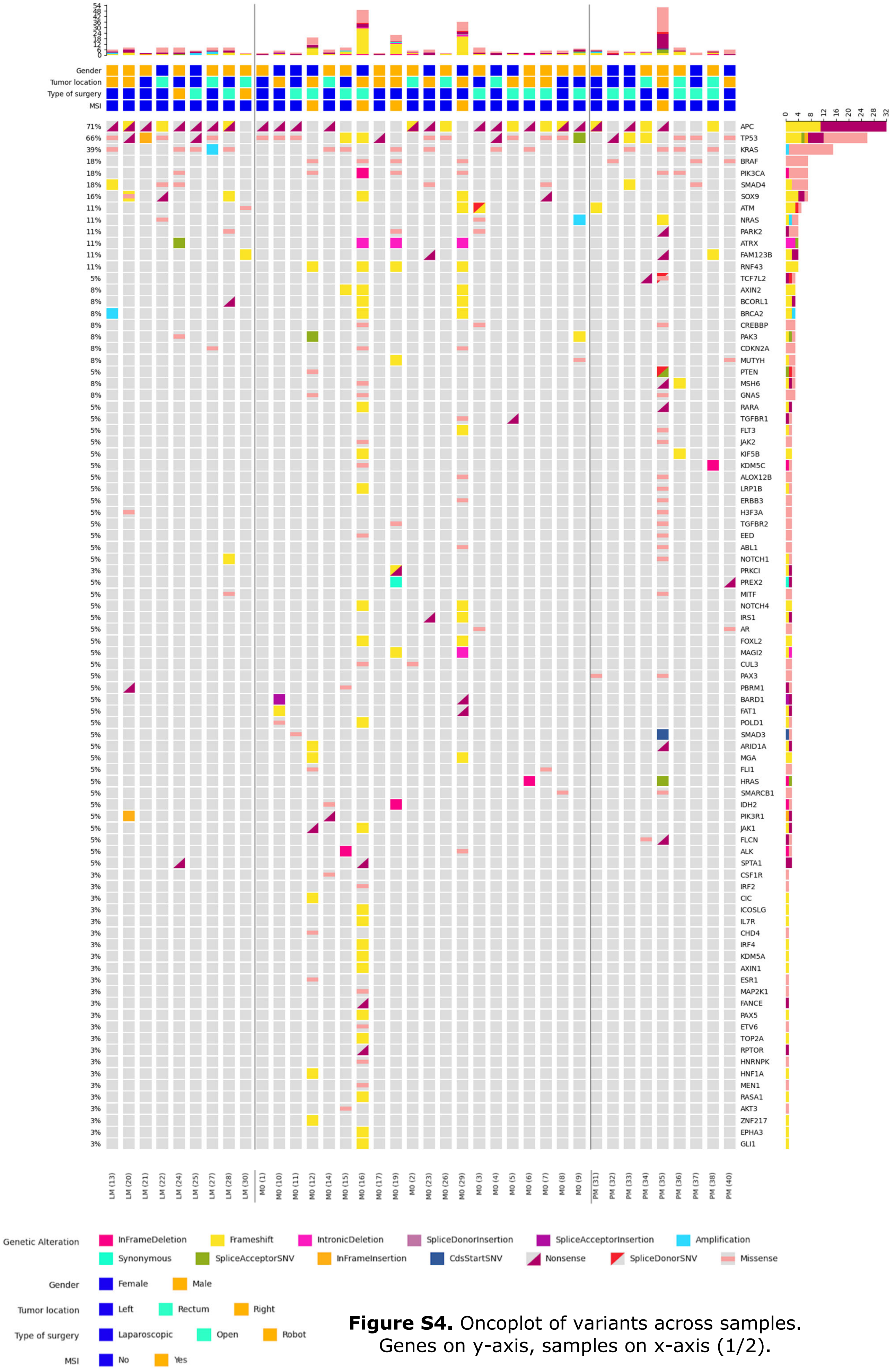

Figure S4. Oncoplot of variants across samples. Genes on y-axis, samples on x-axis (1/2).

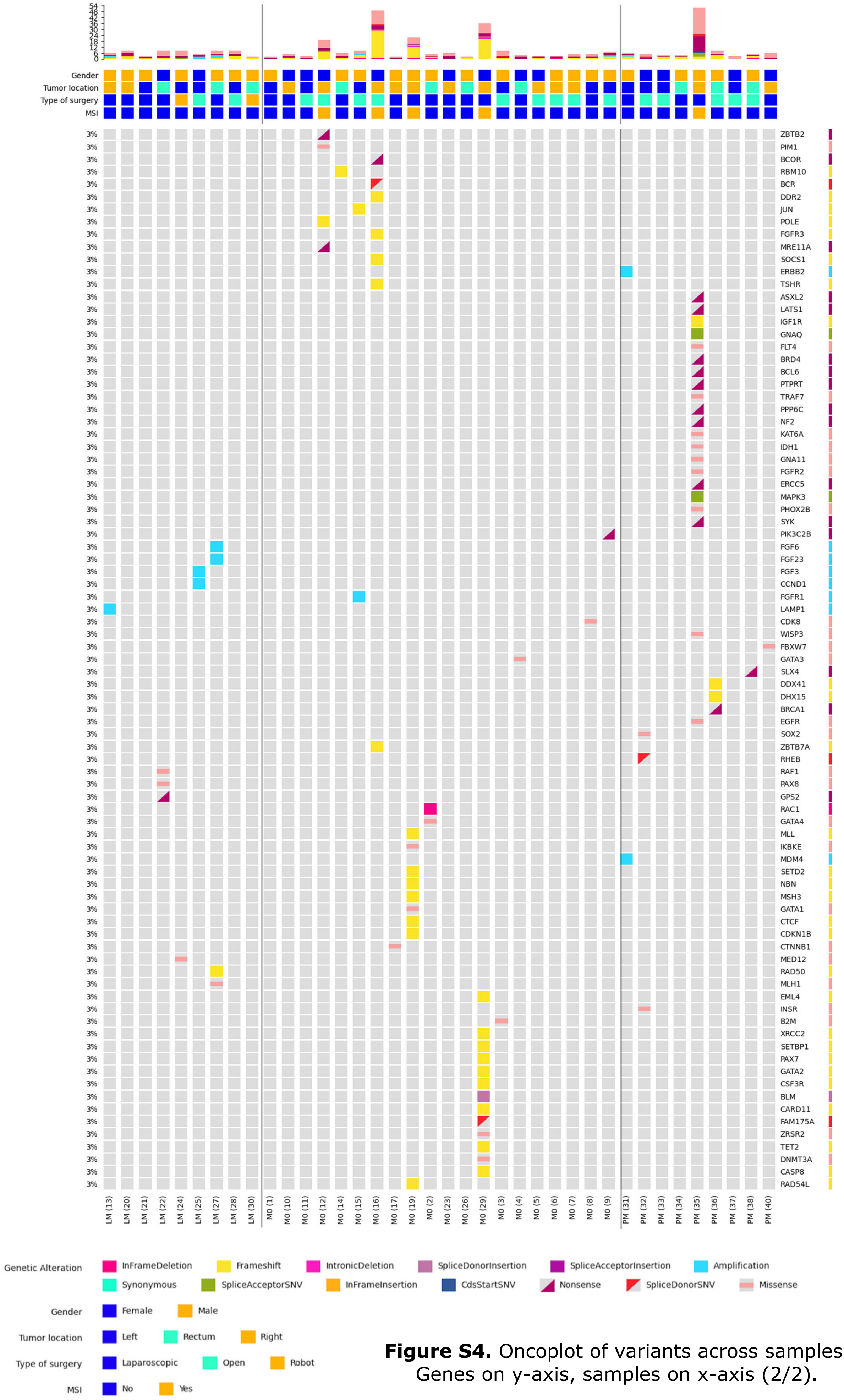

Figure S4. Oncoplot of variants across samples. Genes on y-axis, samples on x-axis (2/2).

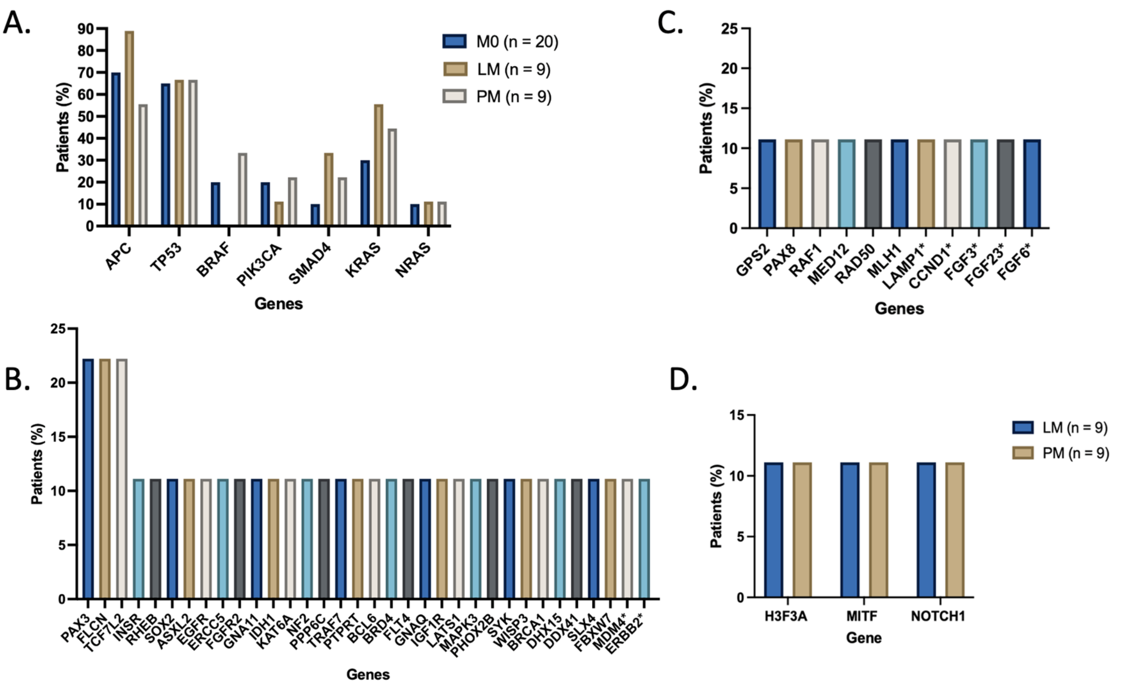

Figure S5. (A) Distribution of well-known oncogenes related to CRC between subgroups. **(B)** Genes mutated or amplified* in PM group only. **(C)** Genes mutated or amplified* in LM group only. **(D)** Genes mutated in LM and PM group (not in M0).

Table S5. Detailed overview percentage of mutated/amplified gene in each subgroup with statistical analysis

Gene	M0 (%) N = 20	LM (%) N = 9	PM (%) N = 9	P value[a]
**APC**	70.0	88.88	55.55	0.331
**TP53**	65.0	66.66	66.66	1.000
**BARD1**	10.0	0.0	0.0	1.000
**FAT1**	10.0	0.0	0.0	1.000
**POLD1**	10.0	0.0	0.0	1.000
**SMAD3**	5.0	0.0	11.11	0.730
**BRAF**	20.0	0.0	33.33	0.219
**ARID1A**	5.0	0.0	11.11	0.730
**CHD4**	5.0	0.0	0.0	1.000
**CIC**	5.0	0.0	0.0	1.000
**ESR1**	5.0	0.0	0.0	1.000
**GNAS**	10.0	0.0	11.11	1.000
**HNF1A**	5.0	0.0	0.0	1.000
**MGA**	10.0	0.0	0.0	1.000
**PIK3CA**	20.0	11.11	22.22	1.000
**PTEN**	5.0	0.0	11.11	0.730
**RNF43**	20.0	0.0	0.0	0.153
**MRE11A**	5.0	0.0	0.0	1.000
**POLE**	5.0	0.0	0.0	1.000
**FLI1**	10.0	0.0	0.0	1.000
**JAK1**	10.0	0.0	0.0	1.000
**PAK3**	10.0	11.11	0.0	1.000
**PIM1**	5.0	0.0	0.0	1.000
**ZBTB2**	5.0	0.0	0.0	1.000

**ZNF217**	5.0	0.0	0.0	1.000
**SMAD4**	10.0	33.33	22.22	0.398
**KRAS**	30.0	55.55	44.44	0.467
**IDH2**	10.0	0.0	0.0	1.000
**CSF1R**	5.0	0.0	0.0	1.000
**PIK3R1**	5.0	11.11	0.0	0.730
**RBM10**	5.0	0.0	0.0	1.000
**AXIN2**	15.0	0.0	0.0	1.000
**PBRM1**	5.0	11.11	0.0	0.730
**AKT3**	5.0	0.0	0.0	1.000
**ALK**	10.0	0.0	0.0	1.000
**JUN**	5.0	0.0	0.0	1.000
**AXIN1**	5.0	0.0	0.0	1.000
**BCOR**	5.0	0.0	0.0	1.000
**BRCA2**	10.0	11.11	0.0	1.000
**CDKN2A**	10.0	11.11	0.0	1.000
**CREBBP**	10.0	0.0	11.11	1.000
**CUL3**	10.0	0.0	0.0	1.000
**ETV6**	5.0	0.0	0.0	1.000
**FANCE**	5.0	0.0	0.0	1.000
**IRF2**	5.0	0.0	0.0	1.000
**JAK2**	5.0	0.0	11.11	0.730
**KDM5C**	5.0	0.0	11.11	0.730
**LRP1B**	5.0	0.0	11.11	0.730
**MEN1**	5.0	0.0	0.0	1.000
**RASA1**	5.0	0.0	0.0	1.000
**SOCS1**	5.0	0.0	0.0	1.000
**SOX9**	15.0	33.33	0.0	0.170
**BCORL1**	10.0	11.11	0.0	1.000
**EPHA3**	5.0	0.0	0.0	1.000
**GLI1**	5.0	0.0	0.0	1.000
**NOTCH4**	10.0	0.0	0.0	1.000
**BCR**	5.0	0.0	0.0	1.000
**DDR2**	5.0	0.0	0.0	1.000
**EED**	5.0	0.0	11.11	0.730
**FGFR3**	5.0	0.0	0.0	1.000
**FOXL2**	10.0	0.0	0.0	1.000
**HNRNPK**	5.0	0.0	0.0	1.000
**ICOSLG**	5.0	0.0	0.0	1.000
**IL7R**	5.0	0.0	0.0	1.000
**IRF4**	5.0	0.0	0.0	1.000
**KDM5A**	5.0	0.0	0.0	1.000
**KIF5B**	5.0	0.0	11.11	0.730
**MAP2K1**	5.0	0.0	0.0	1.000
**MSH6**	5.0	0.0	22.22	0.267
**PAX5**	5.0	0.0	0.0	1.000
**RARA**	5.0	0.0	11.11	0.730
**RPTOR**	5.0	0.0	0.0	1.000
**SPTA1**	5.0	11.11	0.0	0.730
**TOP2A**	5.0	0.0	0.0	1.000
**TSHR**	5.0	0.0	0.0	1.000
**ZBTB7A**	5.0	0.0	0.0	1.000
**ATRX**	15.0	11.11	0.0	0.792
**CTNNB1**	5.0	0.0	0.0	1.000

**CDKN1B**	5.0	0.0	0.0	1.000
**CTCF**	5.0	0.0	0.0	1.000
**GATA1**	5.0	0.0	0.0	1.000
**MSH3**	5.0	0.0	0.0	1.000
**MUTYH**	10.0	0.0	11.11	1.000
**NBN**	5.0	0.0	0.0	1.000
**SETD2**	5.0	0.0	0.0	1.000
**TGFBR2**	5.0	0.0	11.11	0.730
**RAD54L**	5.0	0.0	0.0	1.000
**IKBKE**	5.0	0.0	0.0	1.000
**MAGI2**	10.0	0.0	0.0	1.000
**MLL**	5.0	0.0	0.0	1.000
**PARK2**	10.0	11.11	11.11	1.000
**PRKCI**	5.0	0.0	0.0	1.000
**PREX2**	5.0	0.0	11.11	0.730
**GATA4**	5.0	0.0	0.0	1.000
**RAC1**	5.0	0.0	0.0	1.000
**H3F3A**	0.0	11.11	11.11	0.218
**GPS2**	0.0	11.11	0.0	0.474
**PAX8**	0.0	11.11	0.0	0.474
**RAF1**	0.0	11.11	0.0	0.474
**NRAS**	10.0	11.11	11.11	1.000
**FAM123B**	5.0	11.11	11.11	0.328
**IRS1**	10.0	0.0	0.0	1.000
**MED12**	0.0	11.11	0.0	0.474
**RAD50**	0.0	11.11	0.0	0.474
**MLH1**	0.0	11.11	0.0	0.474
**MITF**	0.0	11.11	11.11	0.218
**NOTCH1**	0.0	11.11	11.11	0.218
**ABL1**	5.0	0.0	11.11	0.730
**ATM**	10.0	11.11	11.11	1.000
**BLM**	5.0	0.0	0.0	1.000
**CASP8**	5.0	0.0	0.0	1.000
**DNMT3A**	5.0	0.0	0.0	1.000
**ERBB3**	5.0	0.0	11.11	0.730
**TET2**	5.0	0.0	0.0	1.000
**ZRSR2**	5.0	0.0	0.0	1.000
**FAM175A**	5.0	0.0	0.0	1.000
**ALOX12B**	5.0	0.0	11.11	0.730
**CARD11**	5.0	0.0	0.0	1.000
**CSF3R**	5.0	0.0	0.0	1.000
**EML4**	5.0	0.0	0.0	1.000
**FLT3**	5.0	0.0	11.11	0.730
**GATA2**	5.0	0.0	0.0	1.000
**PAX7**	5.0	0.0	0.0	1.000
**SETBP1**	5.0	0.0	0.0	1.000
**TGFBR1**	10.0	0.0	0.0	1.000
**XRCC2**	5.0	0.0	0.0	1.000
**AR**	5.0	0.0	11.11	0.730
**B2M**	5.0	0.0	0.0	1.000
**PAX3**	0.0	0.0	22.22	0.102
**INSR**	0.0	0.0	11.11	0.474
**RHEB**	0.0	0.0	11.11	0.474
**SOX2**	0.0	0.0	11.11	0.474

**FLCN**	0.0	0.0	22.22	0.102
**TCF7L2**	0.0	0.0	22.22	0.102
**ASXL2**	0.0	0.0	11.11	0.474
**EGFR**	0.0	0.0	11.11	0.474
**ERCC5**	0.0	0.0	11.11	0.474
**FGFR2**	0.0	0.0	11.11	0.474
**GNA11**	0.0	0.0	11.11	0.474
**IDH1**	0.0	0.0	11.11	0.474
**KAT6A**	0.0	0.0	11.11	0.474
**NF2**	0.0	0.0	11.11	0.474
**PPP6C**	0.0	0.0	11.11	0.474
**SMARCB1**	5.0	0.0	11.11	0.730
**TRAF7**	0.0	0.0	11.11	0.474
**PTPRT**	0.0	0.0	11.11	0.474
**BCL6**	0.0	0.0	11.11	0.474
**BRD4**	0.0	0.0	11.11	0.474
**FLT4**	0.0	0.0	11.11	0.474
**GNAQ**	0.0	0.0	11.11	0.474
**HRAS**	5.0	0.0	11.11	0.730
**IGF1R**	0.0	0.0	11.11	0.474
**LATS1**	0.0	0.0	11.11	0.474
**MAPK3**	0.0	0.0	11.11	0.474
**PHOX2B**	0.0	0.0	11.11	0.474
**SYK**	0.0	0.0	11.11	0.474
**WISP3**	0.0	0.0	11.11	0.474
**BRCA1**	0.0	0.0	11.11	0.474
**DHX15**	0.0	0.0	11.11	0.474
**DDX41**	0.0	0.0	11.11	0.474
**SLX4**	0.0	0.0	11.11	0.474
**GATA3**	5.0	0.0	0.0	1.000
**FBXW7**	0.0	0.0	11.11	0.474
**CDK8**	5.0	0.0	0.0	1.000
**PIK3C2B**	5.0	0.0	0.0	1.000
**LAMP1**	0.0	11.11	0.0	0.474
**FGFR1**	5.0	0.0	0.0	1.000
**CCND1**	0.0	11.11	0.0	0.474
**FGF3**	0.0	11.11	0.0	0.474
**FGF23**	0.0	11.11	0.0	0.474
**FGF6**	0.0	11.11	0.0	0.474
**MDM4**	0.0	0.0	11.11	0.474
**ERBB2**	0.0	0.0	11.11	0.474

[a]Fisher-Freeman-Halton Exact test

References

1. Mi H, Ebert D, Muruganujan A, Mills C, Albou L-P, Mushayamaha T, et al. PANTHER version 16: a revised family classification, tree-based classification tool, enhancer regions and extensive API. Nucleic Acids Research. 2020;49(D1):D394-D403.
2. The Gene Ontology resource: enriching a GOld mine. Nucleic Acids Res. 2021;49(D1):D325-d34.
3. Ashburner M, Ball CA, Blake JA, Botstein D, Butler H, Cherry JM, et al. Gene Ontology: tool for the unification of biology. Nature Genetics. 2000;25(1):25-9.
